# Key residues in TLR4-MD2 tetramer formation identified by free energy simulations

**DOI:** 10.1371/journal.pcbi.1007228

**Published:** 2019-10-14

**Authors:** Alireza Tafazzol, Yong Duan

**Affiliations:** Department of Biomedical Engineering and Genome Center, University of California, Davis, Davis, California, United States of America; University of Houston, UNITED STATES

## Abstract

Toll-like receptors (TLRs) play a central role in both the innate and adaptive immune systems by recognizing pathogen-associated molecular patterns and inducing the release of the effector molecules of the immune system. The dysregulation of the TLR system may cause various autoimmune diseases and septic shock. A series of molecular dynamics simulations and free energy calculations were performed to investigate the ligand-free, lipopolysaccharide (LPS)-bound, and neoseptin3-bound (TLR4-MD2)_2_ tetramers. Compared to earlier simulations done by others, our simulations showed that TLR4 structure was well maintained with stable interfaces. Free energy decomposition by molecular mechanics Poisson-Boltzmann surface area (MM-PBSA) method suggests critical roles that two hydrophobic clusters I_85_-L_87_-P_88_ and I_124_-L_125_-P_127_ of MD2, together with LPS and neoseptin3, may play in TLR4 activation. We propose that 1) direct contacts between TLR4 convex surface and LPS and neoseptin3 at the region around L_442_ significantly increase the binding and 2) binding of LPS and neoseptin3 in the central hydrophobic cavity of MD2 triggers burial of F_126_ and exposure of I_85_-L_87_-P_88_ that facilitate formation of (TLR4-MD2)_2_ tetramer and activation of TLR4 system.

## Author summary

Toll-like receptors (TLRs) play a central role in both the innate and adaptive immune systems and its dysregulation may cause a host of serious and often life-threatening diseases. A great deal has been known about this system. Yet, how exactly this system works and which part is responsible to activate the system remains elusive. This work seeks to identify the key parts of the system that play roles in initiating the signaling cascade. The knowledge gained from this study is expected to shed light to how this important system works which in turn may help design more effective and life-saving anti-inflammatory and anti-cancer drugs.

## Introduction

Toll-like receptor 4 (TLR4) is one of the key initiators of the innate immune response and promotes adaptive immunity [1, 2]. TLR4 belongs to the type I transmembrane proteins and consists of an extracellular ligand-binding domain (ECD; also known as ectodomain) containing leucine rich repeats that folds into a characteristic solenoid horseshoe-like structure; a single transmembrane *α*-helix; and a globular intracellular Toll-interleukin I receptor (TIR) signaling domain, which is responsible for the downstream signaling [3]. Engagement of TLR4-MD2 system by its ligands causes dimerization of the receptor system [3–5] that results in conformational changes within the TIR domains, stabilizing the receptor complex and leading to recruitment of intracellular TIR domain-containing adaptor proteins to initiate downstream signaling cascades [3].

TLR4 is the signaling receptor for lipopolysaccharide (LPS) [6–8] that requires myeloid differentiation factor 2 (MD2) as an LPS co-receptor [9]. Disregulation of the TLR4 system may cause various autoimmune diseases and septic shock [10], whereas sepsis and septic shock accounts for millions of deaths worldwide every year and is the number one cause of death in intensive care units [11]. LPS or endotoxin, found in the outer membrane of Gram-negative bacteria [10], is one of the most powerful immunostimulators known and is responsible for the Salmonella food poisoning and the dangerous endotoxic shock, a severe inflammatory disease that leads rapidly to multi organ failure and death that accounts for about 200,000 deaths per annum in Europe [6]. Studies are also producing interesting findings that TLR4-MD2-LPS activation could provide a link between inflammation with cancer [12–16]. Moreover, A number of studies suggest a possible role for TLR4 in cardiovascular disease [17, 18], inflammatory bowel disease [19], HIV-1 disease [20], Alzheimer’s disease [21], rheumatoid arthritis [22, 23], renal disease [24], obesity, and diabetes types I and II [25]. Thus, understanding the mechanism of action of LPS-mediated immune activation, in particular to TLR4 system, is an important objective in medical research. To this end, although several anti-inflammatory compounds and antibodies modulating TLR4 have already undergone preclinical and clinical evaluations [12, 26–29] and have reached clinical trials for various indications [30–33], finding an effective TLR4 or/and MD2 modulator remains a challenging endeavor. Hence, elucidation of the mechanism of the TLR4-MD2 system has high potential for the design of new molecules able to modulate TLR4 immune response.

Although neoseptin3 [34], a chemically synthesized peptidomimetic compound, shares no structural similarity to LPS, it can also bind and activate the mouse TLR4-MD2 complex [35] with an EC_50_ of 18.5 μM. Thus, non-LPS ligands of natural origin might be capable of activating the TLR4-MD2 complex without an LPS-like structure. The relatively modest potency of neoseptin3 and the nano-mole level high potency of LPS provide an avenue to evaluate the underlying binding and activation mechanisms in terms of their commonality and differences that may in turn help the discovery of antagonistic compounds.

In this work, our goal is to study the key ligand-receptor interactions to rationalize the mechanism for TLR4-MD2 modulation by computational techniques to gain insights into the mechanism behind the TLR4-MD2 activation and into the molecular recognition process at the atomic level. Our study will complete a missing point of dimerization and how TLR4-MD2 system binds to its natural and synthetic ligands and how these binding interfaces lead to TLR4 dimerization that triggers activation of downstream signaling.

Recently, Huber et al. [36] developed near-atomic computational models to simulate LPS transfer through the TLR4 pathway and revealed that LPS recognition is favored by a thermodynamic funnel of increasing affinity along the proposed transfer pathway via CD14 to the TLR4-MD2 complex. They also proposed the role of F_126_ in MD2 as a key mediator in the microscopic LPS transfer between CD14 and TLR4-MD2 complex. Moreover, their energy calculations for the binding of ligand-free, apo MD2 to TLR4 showed that heterodimeric TLR4-MD2 complex exists in a pre-assembled state, consistent with the single-molecule studies of Ryu et al. [37] in which LPS transfer to MD2 was primarily TLR4 dependent, and with experimental demonstrations of stronger affinity for LPS binding to the TLR4-MD2 complex than to MD2 [38].

In recent years, crystallographic data [35, 39] have illuminated the structural features of TLR4-MD2 system and revealed that: 1) overall complex organization is highly conserved and 2) the ligand-bound tetramer undergoes local structural changes in F_126_ of MD2. Crystallography studies have summarized comprehensively the structural differences induced by ligand binding. In this work, the TLR4-MD2 system is examined systematically by free energy calculations which provides a qualitative picture on the key factors driving and stabilizing the tetramer formation.

## Results and discussion

### Stable complexes observed in simulations

We have performed series of molecular dynamics (MD) simulations on four mouse TLR4-MD2 complexes [35]: TLR4-MD2 heterodimer, (TLR4-MD2)_2_ tetramer, LPS-bound (TLR4-MD2)_2_ tetramer, neoseptin3-bound (TLR4-MD2)_2_ tetramer using AMBER simulation package [40, 41]. Each system was simulated four times to allow an assessment of the consistency of the observations. Each simulation was performed to 1.2 μs, in a fully solvated periodic box of water. Overall, all simulations were stable, as measured by the C_α_ root mean-square difference (RMSD) from their respective X-ray structures after rigid-body alignment (S1 Table). Among the four systems, the largest average RMSD (over the last 1.0 μs and four trajectories) was 3.93 Å, from the ligand-free tetramer. The smallest average RMSD was 2.51 Å, from the LPS-bound tetramer. In fact, all 8 ligand-bonded simulations exhibited higher stability than the ligand-free tetramer and only one of the four (TLR4-MD2-neoseptin3)_2_ trajectory had slightly elevated dynamics with an average C_α_ RMSDs exceeded 3.0 Å; all other 7 ligand-bonded trajectories had average C_α_ RMSDs below 3.0 Å (S1 Table). In comparison, of the 8 ligand-free simulations, only one of the four TLR4-MD2 heterodimer trajectories had the average C_α_ RMSDs smaller than 3.0 and all other 7 ligand-free trajectories had the average C_α_ RMSDs exceeded 3.0 Å (S1 Table). The larger RMSDs of the ligand-free systems are consistent with the observation that TLR4-MD2 does not form stable tetramer under physiological concentration.

TLR4 has been an extensively studied system. Recent computational studies included molecular dynamics simulations of de Aguiar et al. [42] on the human TLR4 complex (PDB code 3FXI) using GROMOS53a6 force field. A highly dynamic (TLR4-MD2-LPS)_2_ complex was observed in their 100 ns simulations with an overall RMSD ranged from about 8 Å to about 12 Å. These are significantly larger than what we observed in our simulations, even though their simulations were more than an order of magnitude shorter than ours. In another recent study, Anwar and Choi [43] also studied the dynamics of wild-type (PDB code 3FXI) and mutated (4G8A) human TLR4-MD2-LPS complexes by 200 ns simulations using the AMBER99SB-ILDN force field parameters. Their observed RMSDs were on the order of 3.5 Å, considerably larger than what we observed in our TLR4-MD2-LPS simulations, even though our simulation times were six times as long, and larger than most of what we observed in this study, and comparable to the level we observed in the ligand-free complex. In fact, in our simulations, the average C_α_ RMSD of TLR4-MD2-LPS complex was 2.5 Å, the smallest among all four complexes we studied and notably smaller than those observed by Anwar and Choi. Thus, our simulations indicate a considerably more stable TLR4-MD2-LPS complex than what Anwar and Choi observed. Paramo et al. [44] also performed 100 ns simulations and noticed that MD2 is highly flexible. In our case, the MD2 structures were well-maintained.

### Analysis of binding free energies

**Fig 1:**
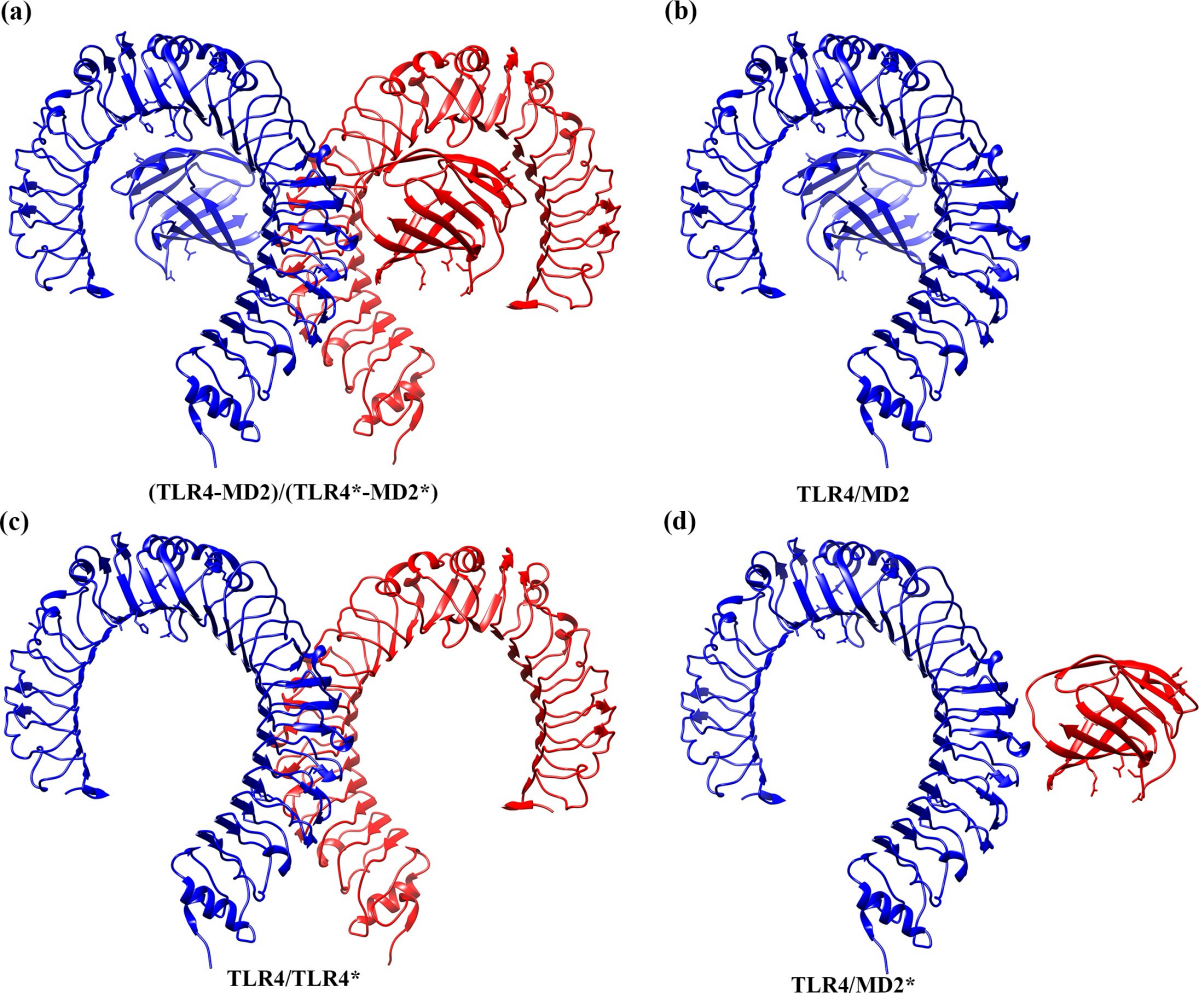
The Illustration of the dimer interfaces in the free energy calculations. **a)** (TLR4-MD2)/(TLR4*-MD2*), **b)** TLR4/MD2, **c)** TLR4/TLR4*, and **d)** TLR4/MD2* interface. The TLR4 and MD2 are colored in blue, and TLR4* and MD2* are colored in red. Displayed images were created with UCSF Chimera software [63].

The tetramer (TLR4-MD2)_2_ complex is organized as a dimer of two symmetric TLR4-MD2 heterodimers (Fig 1A). For clarity, we denote the four monomers in the tetramer as TLR4, MD2, TLR4*, and MD2* in which TLR4 and MD2 form a heterodimer and TLR4* and MD2* form the other heterodimer. Since the two MD2 subunits in the tetramer are not in direct contacts, there are three distinct dimer interfaces between the monomers in the complex, namely, TLR4/MD2 (and its symmetry-related image TLR4*/MD2*), TLR4/TLR4*, and TLR4/MD2* (and its symmetry-related image TLR4*/MD2) (Fig 1). We evaluated the binding free energies of all these interfaces as well as that of the (TLR4-MD2)/(TLR4*-MD2*) interface (Fig 1A) with both molecular mechanics generalized Born surface area (MM-GBSA) and molecular mechanics Poisson-Boltzmann surface area (MM-PBSA) methods (Tables 1, 2 and S2–S7). A total of 4000 frames for each system were used in the free energy calculations (1000 frames from the last 1.0 μs of each MD trajectory with an interval of 1.0 ns). The binding free energies averaged over the individual trajectories are listed in S2–S6 Tables and their averages of different trajectories are listed in Table 1; the negative values are a favorable free energy, while positive values are an unfavorable. Similarly, the (TLR4-MD2)/(TLR4*-MD2*) binding free energies of the individual trajectories are in S7 Table and the averages over different trajectories are in Table 2. There is a good agreement between binding free energies Δ*G* estimated by MM-GBSA and MM-PBSA methods and same trends are observed in both methods when comparing free energies of the ligand-free and ligand-bound complexes in these interfaces. We will focus our discussions based on the MM-PBSA results.

**Table 1:** The binding free energies (Δ*G*) in kcal/mol at the five interfaces computed by both molecular mechanics generalized Born surface area (MM-GBSA) and molecular mechanics Poisson-Boltzmann surface area (MM-PBSA) methods. These interfaces are TLR4/MD2 (and its symmetry-related image TLR4*/MD2*), TLR4/TLR4*, and TLR4/MD2* (and its symmetry-related images TLR4*/MD2). The free energies in each of the ligand-free TLR4-MD2 heterodimer, (TLR4-MD2)_2_ tetramer, lipopolysaccharide (LPS)-bound (TLR4-MD2)_2_ tetramer, and neoseptin3-bound (TLR4-MD2)_2_ tetramer complexes are averaged over the average of their 4 different trajectories consisting of overall 4000 frames. A negative value is a favorable free energy, while a positive value is an unfavorable. The values in parenthesis are standard deviation. Δ*E*_*MM*_ is molecular mechanics free energy which is divided into Δ*E*_*ele*_ and Δ*E*_*vdw*_ representing the contributions from the electrostatic and van der Waals interactions, respectively. Δ*G*_*sol*_ is solvation free energy expressed by Δ*G*_*pol*_ and Δ*G*_*nonpol*_, the polar and non-polar contributions, respectively.

			Generalized Born (GB)			Poisson-Boltzmann (PB)		
Complex	Δ*E*_*MM*_		Δ*G*_*sol*_		Δ*G*	Δ*G*_*sol*_		Δ*G*
	Δ*E*_*ele*_	Δ*E*_*vdw*_	Δ*G*_*pol*_	Δ*G*_*nonpol*_		Δ*G*_*pol*_	Δ*G*_*nonpol*_	
**TLR4/MD2 Interface**								
TLR4-MD2	-605.08 (25.29)	-127.33 (7.47)	636.34 (29.97)	-20.18 (1.43)	-116.23 (9.82)	622.87 (30.70)	-14.99 (0.88)	-124.51 (3.39)
(TLR4-MD2)_2_	-576.73 (35.41)	-103.29 (3.01)	593.37 (27.83)	-16.66 (0.72)	-103.28 (11.80)	582.83 (33.70)	-11.89 (0.24)	-109.06 (6.36)
(TLR4-MD2-LPS)_2_	-581.94 (18.00)	-116.00 (3.05)	610.25 (14.12)	-18.56 (0.61)	-106.23 (7.88)	600.95 (16.59)	-13.18 (0.30)	-110.15 (5.74)
(TLR4-MD2-neoseptin3)_2_	-482.09 (28.67)	-108.23 (2.97)	505.75 (29.02)	-16.79 (0.24)	-101.35 (3.77)	491.42 (29.40)	-11.68 (0.09)	-110.57 (3.13)
TLR4*/MD2* Interface								
(TLR4-MD2)_2_	-444.92 (43.23)	-100.61 (5.29)	473.45 (38.53)	-15.74 (0.90)	-87.80 (8.07)	458.60 (39.29)	-11.62 (0.36)	-98.53 (4.82)
(TLR4-MD2-LPS)_2_	-561.04 (15.54)	-116.12 (3.18)	588.92 (8.40)	-18.31 (0.40)	-106.54 (5.70)	575.68 (10.43)	-12.77 (0.28)	-114.24 (3.60)
(TLR4-MD2-neoseptin3)_2_	-520.02 (46.96)	-107.71 (1.39)	543.03 (42.85)	-16.82 (0.57)	-101.50 (6.12)	526.70 (43.23)	-11.88 (0.44)	-112.90 (5.79)
TLR4/TLR4* Interface								
(TLR4-MD2)_2_	-125.77 (80.25)	-42.38 (7.92)	158.01 (83.08)	-7.56 (1.59)	-17.67 (7.24)	134.62 (84.09)	-6.35 (1.34)	-39.85 (5.01)
(TLR4-MD2-LPS)_2_	242.32 (15.64)	-69.91 (4.84)	-163.36 (18.11)	-9.40 (0.86)	-0.35 (3.94)	-190.45 (17.64)	-8.61 (0.59)	-26.65 (4.01)
(TLR4-MD2-neoseptin3)_2_	242.08 (34.40)	-81.08 (8.24)	-161.06 (38.64)	-10.48 (1.64)	-10.53 (5.54)	-189.76 (37.83)	-9.00 (1.20)	-37.76 (6.70)
TLR4/MD2* Interface								
(TLR4-MD2)_2_	-34.85 (28.56)	-40.48 (10.29)	60.34 (29.32)	-5.43 (1.26)	-20.41 (6.64)	50.00 (29.75)	-5.08 (1.16)	-30.40 (6.14)
(TLR4-MD2-LPS)_2_	-167.40 (65.22)	-74.10 (2.61)	208.88 (53.56)	-9.95 (0.36)	-42.54 (11.17)	203.08 (51.21)	-7.99 (0.23)	-46.38 (14.46)
(TLR4-MD2-neoseptin3)_2_	-120.33 (58.50)	-75.74 (4.41)	160.46 (51.20)	-9.85 (0.84)	-45.44 (10.86)	152.70 (50.86)	-7.74 (0.51)	-51.10 (10.26)
TLR4*/MD2 Interface								
(TLR4-MD2)_2_	-117.32 (14.28)	-52.39 (9.84)	152.39 (16.31)	-7.05 (1.26)	-24.35 (6.36)	140.81 (17.21)	-6.44 (1.05)	-35.32 (5.22)
(TLR4-MD2-LPS)_2_	-173.90 (67.59)	-73.27 (4.63)	212.96 (55.82)	-9.77 (1.14)	-43.96 (15.91)	204.46 (54.31)	-7.86 (0.66)	-50.56 (17.49)
(TLR4-MD2-neoseptin3)_2_	-175.57 (33.84)	-77.57 (9.59)	215.18 (25.08)	-10.30 (1.19)	-48.24 (5.13)	203.74 (25.37)	-8.06 (0.70)	-57.44 (3.98)

❖ The four monomers in the tetramers are denoted as TLR4, MD2, TLR4*, and MD2* in which TLR4 and MD2 form a heterodimer and TLR4* and MD2* form the other heterodimer.

**Table 2:** The binding free energies (Δ*G*) in kcal/mol between TLR4-MD2 and TLR4*-MD2* at the (TLR4-MD2)/(TLR4*-MD2*) interface computed by both molecular mechanics generalized Born surface area (MM-GBSA) and molecular mechanics Poisson-Boltzmann surface area (MM-PBSA) methods. The free energies in each of the ligand-free (TLR4-MD2)_2_ tetramer, lipopolysaccharide (LPS)-bound (TLR4-MD2)_2_ tetramer, and neoseptin3-bound (TLR4-MD2)_2_ tetramer complexes are averaged over the average of their 4 different trajectories consisting of overall 4000 frames. A negative value is a favorable free energy, while a positive value is an unfavorable. The values in parenthesis are standard deviation. Δ*E*_*MM*_ is molecular mechanics free energy which is divided into Δ*E*_*ele*_ and Δ*E*_*vdw*_ representing the contributions from the electrostatic and van der Waals interactions, respectively. Δ*G*_*sol*_ is solvation free energy expressed by Δ*G*_*pol*_ and Δ*G*_*nonpol*_, the polar and non-polar contributions, respectively.

			Generalized Born (GB)			Poisson-Boltzmann (PB)		
Complex	Δ*E*_*MM*_		Δ*G*_*sol*_		Δ*G*	Δ*G*_*sol*_		Δ*G*
	Δ*E*_*ele*_	Δ*E*_*vdw*_	Δ*G*_*pol*_	Δ*G*_*nonpol*_		Δ*G*_*pol*_	Δ*G*_*nonpol*_	
(TLR4-MD2)/(TLR4*-MD2*) Interface								
(TLR4-MD2)_2_	-278.97 (63.65)	-135.26 (23.46)	370.21 (70.03)	-20.04 (3.29)	-64.04 (12.45)	327.11 (73.18)	-17.88 (2.82)	-104.98 (9.45)
(TLR4-MD2-LPS)_2_	-96.35 (96.03)	-217.32 (8.54)	252.09 (79.06)	-29.06 (1.60)	-90.63 (19.49)	214.67 (71.48)	-24.23 (1.00)	-123.22 (26.33)
(TLR4-MD2-neoseptin3)_2_	-43.43 (71.02)	-234.42 (13.96)	202.22 (64.14)	-30.62 (2.40)	-106.24 (12.67)	157.17 (61.25)	-24.78 (1.34)	-145.44 (13.76)

❖ The four monomers in the tetramers are denoted as TLR4, MD2, TLR4*, and MD2* in which TLR4 and MD2 form a heterodimer and TLR4* and MD2* form the other heterodimer.

Compared to the TLR4/MD2 and TLR4*/MD2* interfaces in tetramer simulations, the TLR4/MD2 interface (Fig 1B) in the heterodimer TLR4-MD2 simulations was the most stable, as judged by its most favorable binding free energy of Δ*G*_*PB*_ = -124.51±3.39 kcal/mol which was more than -10 kcal/mol more stable than the TLR4/MD2 and TLR4*/MD2* interfaces observed in the tetramers as shown in Tables 1, S2 and S3. When bound to the ligands, the binding free energies of the heterodimer TLR4/MD2 and TLR4*/MD2* interfaces in (TLR4-MD2-LPS)_2_ tetramer are Δ*G*_*PB*_ = -110.15±5.74 and -114.24±3.60 kcal/mol, respectively. In (TLR4-MD2-neoseptin3)_2_ tetramer, they are Δ*G*_*PB*_ = -110.57±3.13 and -112.90±5.79 kcal/mol, respectively. In the absence of ligands, binding free energies of the same interfaces in the ligand-free tetramer (TLR4-MD2)_2_ are Δ*G*_*PB*_ = -109.06±6.36 and -98.53±4.82 kcal/mol, respectively. In fact, the binding free energies of the heterodimer TLR4/MD2 interface remain essentially the same in all three tetramer (TLR4-MD2)_2_ complexes, regardless of ligand-binding state. The same trend is observed with Δ*G*_*GB*_. Consistently, the heterodimer TLR4/MD2 interface is notably weakened by formation of the tetramer complex, irrespective to the binding of ligands or not. In fact, binding of ligands or not has no significant effect to this particular interface.

The interface between the two heterodimers (TLR4-MD2) and (TLR4*-MD2*) can be viewed as three separate interfaces: TLR4/TLR4*, TLR4/MD2*, and TLR4*/MD2. Among them, the TLR4/MD2* and TLR4*/MD2 are symmetry-related. Ligand binding has mixed effects to the TLR4/TLR4* interface; it is notably weakened in (TLR4-MD2-LPS)_2_ by 13 kcal/mol and slightly weakened in (TLR4-MD2-neoseptin3)_2_ by about 2.0 kcal/mol (Tables 1 and S4).

On the other hand, ligand binding makes the TLR4/MD2* and its symmetry-related image TLR4*/MD2 interfaces consistently more favorable (Tables 1, S5 and S6). Relative to the ligand-free complex, binding of LPS and neoseptin3 strengthened the TLR4/MD2* interface by -16 kcal/mol (LPS) to -21 kcal/mol (neoseptin3) and the TLR4*/MD2 interface by -15 kcal/mol (LPS) to -22 kcal/mol (neoseptin3). If we add these terms together, the combined contribution of TLR4/MD2*, TLR4*/MD2, and TLR4/TLR4* interfaces in each complex would have made the (TLR4-MD2)/(TLR4*-MD2*) binding more favorable by -18.8 kcal/mol and -39.3 kcal/mol, for (TLR4-MD2-LPS)_2_ and (TLR4-MD2-neoseptin3)_2_, respectively. Indeed, the (TLR4-MD2)/(TLR4*-MD2*) interface in the ligand-bound complexes was also significantly stronger than in the ligand-free complex (Tables 2 and S7) and was -18.2 kcal/mol more favorable in (TLR4-MD2-LPS)_2_ complex and -40.5 kcal/mol in (TLR4-MD2-neoseptin3)_2_; both are close to their respective calculated binding free energies by addition of binding free energy of the individual interfaces. Thus, the calculation suggests that the binding effects of the ligands LPS and neoseptin3 in (TLR4-MD2)/(TLR4*-MD2*) are primarily additive. This is understandable because these interfaces are spatially separated. Judging from the fact that binding of the ligands makes the TLR4/MD2* and TLR4*/MD2 significantly stronger by -15 (LPS) to -22 (neoseptin3) kcal/mol and has minor effect on the TLR4/TLR4* interface, we conclude that binding of LPS or neoseptin3 stabilizes tetramer (TLR4-MD2)_2_ complex primarily by inducing stronger binding at the TLR4/MD2* and TLR4*/MD2 interfaces.

It is also interesting to note that the TLR4/MD2* and TLR4*/MD2 interfaces are stronger when bound to neoseptin3 than LPS. As a consequence, the (TLR4-MD2)/(TLR4*-MD2*) interface is notably stronger in neoseptin3 complex than in LPS complex. Remarkably, this is achieved in neoseptin3 with minor changes to the binding free energy at the TLR4/TLR4* interface (weakened by 2 kcal/mol) whereas the same interface was weakened by more than 13 kcal/mol in LPS complex (Tables 1 and S4).

Binding of two constituents often involves formation of contacts and loss of solvent-exposed surface areas. This can be evaluated by decomposing the free energies into two, often compensating contributions: interaction energy and desolvation free energy. In the absence of ligands, the TLR4/TLR4* interface has favorable interaction energy of -168 kcal/mol, dominated by the strong electrostatic energy of -126 kcal/mol (Table 1). This favorable interaction energy is compensated by 128 kcal/mol desolvation free energy (Table 1), indicating that formation of TLR4/TLR4* interface, in the absence of ligands, is primarily driven by enthalpy, mainly the electrostatic force. In contrast, compared to the tetramer without the ligands, both LPS and neoseptin3 increases the electrostatic interaction energies about 368 kcal/mol at the TLR4/TLR4* interface (Table 1). Those unfavorable interaction energies were compensated by the favorable solvation free energies by -327 kcal/mol in both cases, compared to the ligand-free tetramer (Table 1). Therefore, with the ligands, formation of the TLR4/TLR4* interface is driven primarily by desolvation and burial of hydrophobic surface, much more so than in the ligand-free tetramer.

The TLR4/MD2* interface has more favorable interaction energies (-166 kcal/mol with LPS and -121 kcal/mol with neoseptin3) than the apo complex without ligands and the solvation free energies are unfavorable by 150 kcal/mol with LPS and 100 kcal/mol with neoseptin3, compared to the ligand-free tetramer (Table 1). Similarly, the TLR4*/MD2 interface has more favorable interaction energies (-77 kcal/mol with LPS and -83 kcal/mol with neoseptin3) than the apo complex and unfavorable solvation free energies (62.23 and 61.31 kcal/mol for LPS- and neoseptin3-bound tetramers, respectively) (Table 1). It is interesting to note that, despite the large differences in the interaction energies between the two interfaces, the binding free energies are remarkably consistent. The stronger interaction energies indicate that, unlike the other two interfaces, binding of LPS and neoseptin3 makes the TLR4/MD2* and TLR4*/MD2 interfaces more energetically favorable. More specifically, formation of TLR4/MD2* and TLR4*/MD2 interfaces is driven by electrostatics and disfavored by solvation.

### Key residues on interfaces identified from per-residue free energy decomposition

**Fig 2:**
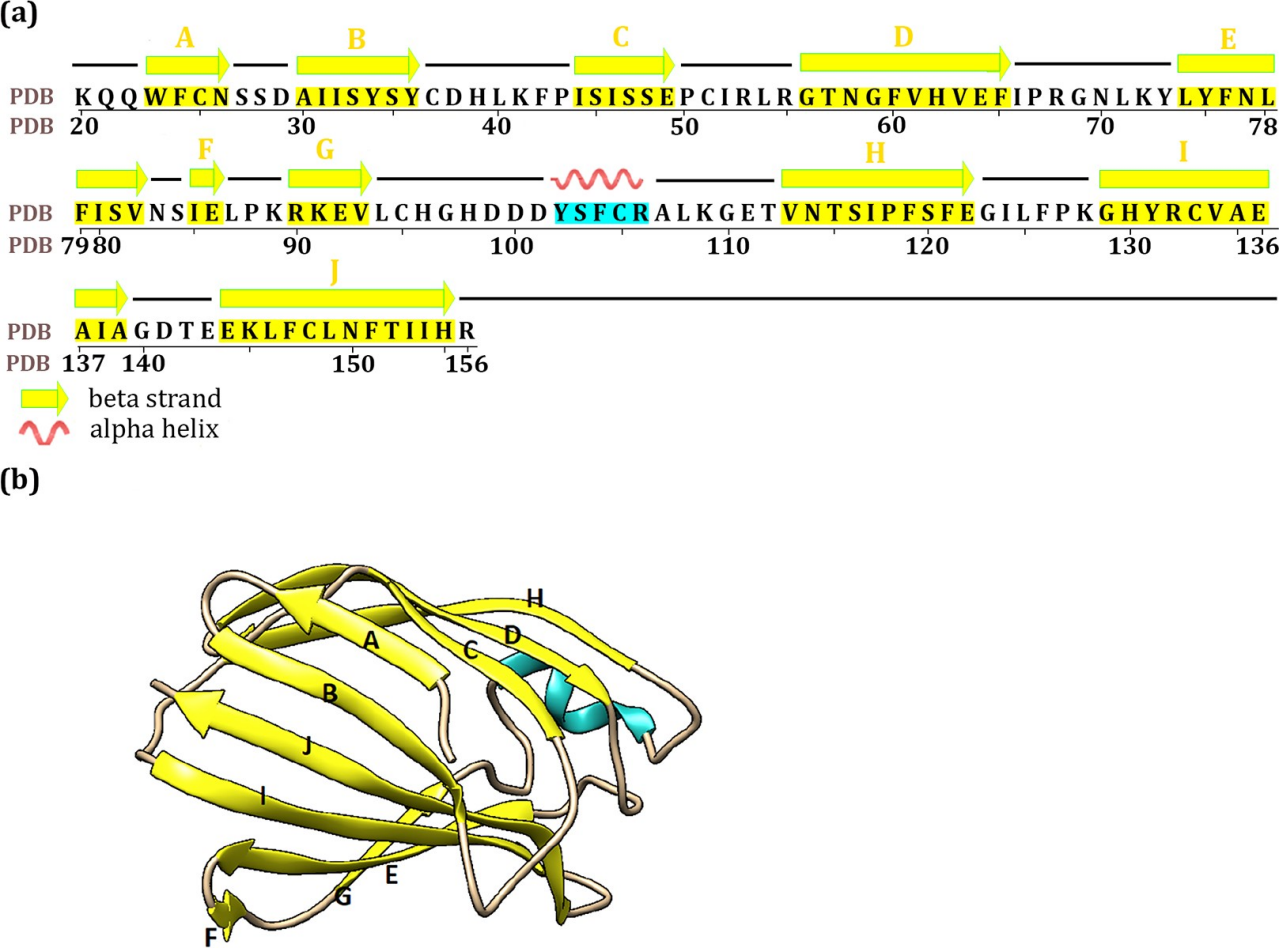
The structure of mouse MD2 in the lipopolysaccharide (LPS)-bound (TLR4-MD2)_2_ tetramer complex. **a)** The residue sequences of MD2 and the residues within secondary structures of β-sheet and helix are colored in yellow and cyan shadow respectively. The C strand in the neoseptin3-bound complex starts at residue S_45_. In the ligand-free complex: the A strand ends at S_27_, the C strand starts at S_45_, the D strand starts at T_57_, the F and G strands are combined together as one strand (i.e., I_85_ to V_93_), the H strand ends at F_121_, and the I strand starts at C_133_. **b)** The three-dimensional crystal structure of MD2. The secondary structures of β-sheet and helix are colored in yellow and cyan respectively.

**Fig 3:**
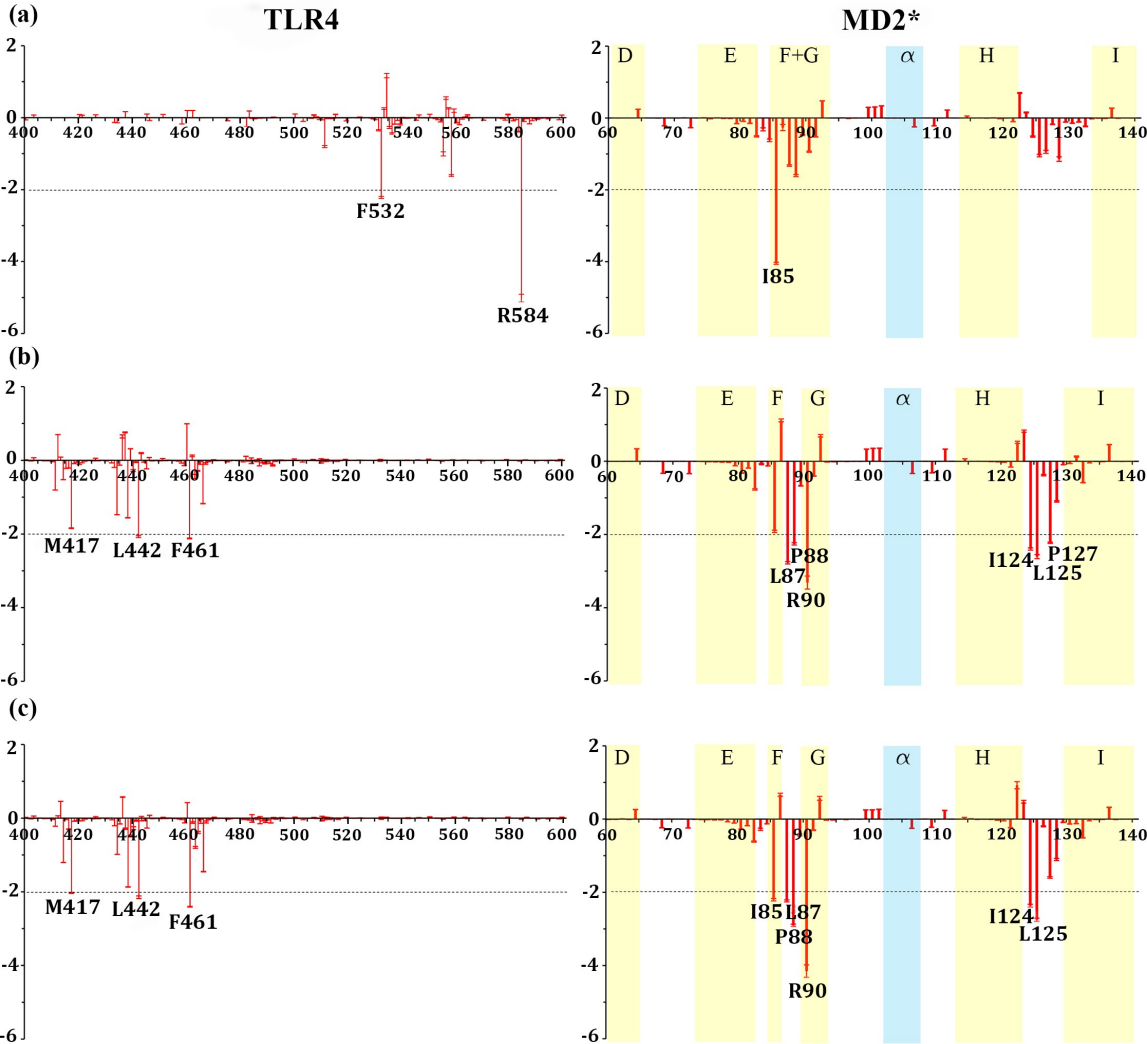
The per residue free energy contribution spectrums of TLR4 and MD2* in the TLR4/MD2* interface. **a)** the ligand-free (TLR4-MD2)_2_ tetramer, **b)** the lipopolysaccharide (LPS)-bound (TLR4-MD2)_2_ tetramer, and **c)** the neoseptin3-bound (TLR4-MD2)_2_ tetramer complex. Only the binding areas are shown with standard errors as red bars, estimated as standard deviation divided by square root of 1000.

To provide a qualitative assessment on the key residues with most contributions in the complex and dimer formation, we decomposed the binding free energies into per-residue contribution for all monomers, TLR4, MD2 (Fig 2), TLR4*, and MD2*, for interfaces in all the 4 systems. The residues with binding free energy contributions lower than -2.0 kcal/mol and greater than 2 kcal/mol are identified as key (favorable) residues and unfavorable residues, respectively, and are listed in Tables 3, S8 and S9. Figs 3 and S1–S9 show the per-residue binding free energy contributions with the key residues highlighted in the respective structures.

**Table 3:** The key residues of TLR4 and MD2 identified by per-residue free energy decomposition (kcal/mol) in the TLR4/MD2* and TLR4*/MD2 interfaces of the ligand-free (TLR4-MD2)_2_ tetramer, lipopolysaccharide (LPS)-bound (TLR4-MD2)_2_ tetramer, and neoseptin3-bound (TLR4-MD2)_2_ tetramer complexes. The values are averaged over the 1000 frames of the combined 4 trajectories of each system. A negative value is a favorable free energy, while a positive value is an unfavorable.

		Complex					
		(TLR4-MD2)_2_		(TLR4-MD2-LPS)_2_		(TLR4-MD2-neoseptin3)_2_	
		Interface					
Monomer	Residue	TLR4/MD2*	TLR4*/MD2	TLR4/MD2*	TLR4*/MD2	TLR4/MD2*	TLR4*/MD2
TLR4	M417	0.00	0.00	-1.84	-1.80	-2.03	-3.14
	L442	0.00	0.00	-2.07	-2.26	-2.15	-2.24
	F461	-0.01	-0.01	-2.12	-2.30	-2.41	-2.31
	W511	-0.81	-2.08	-0.01	-0.01	-0.01	-0.01
	F532	-2.22	-2.02	0.00	0.00	0.00	0.00
	R584	-5.02	-3.52	-0.02	0.00	-0.03	-0.02
MD2	R55	-0.46	-0.59	-0.80	-0.82	-0.71	-3.21
	I85	-4.05	-3.95	-1.91	-2.12	-2.20	-2.05
	L87	-1.33	-1.90	-2.77	-2.56	-2.24	-2.74
	P88	-1.60	-1.61	-2.26	-2.85	-2.90	-2.22
	R90	-0.94	-1.13	-3.31	-4.40	-4.15	-3.57
	I124	-0.52	-1.55	-2.40	-2.89	-2.37	-3.16
	L125	-1.05	-2.00	-2.61	-2.99	-2.71	-3.78
	F126	-0.94	-1.59	-0.38	-0.48	-0.20	-0.22
	P127	-0.18	-0.25	-2.23	-2.17	-1.60	-1.47
	K128	-1.14	-0.56	-1.10	-1.07	-1.10	-1.35
	H155	0.34	-0.05	-0.07	-0.08	-0.08	-0.13
Ligand	LPS	–	–	-4.75	-4.90	–	–
	neoseptin3	–	–	–	–	-4.86	-4.47

❖ The four monomers in the tetramers are denoted as TLR4, MD2, TLR4*, and MD2* in which TLR4 and MD2 form a heterodimer and TLR4* and MD2* form the other heterodimer.

A remarkable similarity is observed in the TLR4/MD2 interface. Across all four complexes, including the heterodimer TLR4-MD2, the interaction patterns are remarkably similar (S1 Fig). The similarity extends to the symmetry-related TLR4*/MD2* interface (S3 Fig). In all 7 cases (S1 and S3 Figs), those contributed the most favorably are essentially the same set of residues and those contributed the most unfavorably are also essentially the same. For example, the most favorable contribution on the TLR4 side comes from R_337_ in all cases. On the MD2 side, the most favorable contributions come from S_103_ and R_106_ in all cases. The most unfavorable contributions are from D_264_ and E_265_ on the TLR4 side and D_99_, D_101_, and E_111_ on the MD2 side. These key residues form two patches on the concave side of TLR4 (S2 and S4 Figs); one is around R_337_ and the other smaller patch is around T_109_. On MD2, most of the key residues are on the helix and its adjacent loops with a small patch around R_68_.

In the TLR4/TLR4* interface, largely due to the fact that ligand binding changes the TLR4/TLR4* interface, there are obvious differences between the ligand-free and ligand-bound complexes whereas the two ligand-bound complexes are themselves similar (S5 Fig). There is also similarity between the two TLR4 monomers in all systems. In the ligand-free complex, the most favorable contributions come from K_433_ and R_434_. But in ligand-bound complexes, K_433_ is one of the two residues that make significant unfavorable contribution (the other is E_507_).

One of the profound changes to the (TLR4-MD2)_2_ tetramer complex upon binding to LPS or neoseptin3 is the significant shift of the binding interface between TLR4 and MD2* as well as the symmetry image TLR4* and MD2. This is clearly shown from the pre-residue binding free energy contributions. In the ligand-free tetramer, (TLR4-MD2)_2_, interactions are primarily around F_532_ and R_584_ of TLR4 between TLR4 and MD2* (Fig 3) as well as between TLR4* and MD2 (S8 Fig). In the ligand-bound tetramers, however, neither F_532_ nor R_584_ makes significant contribution (Fig 3 and Table 3). Instead, the most favorable contributions come from M_417_, L_442_, and F_461_ of TLR4 (Fig 3). Interestingly, the changes on the MD2 side is notably less profound and the residues that make the most favorable contributions form two patches in all three complexes, with the primary patch around I_85_/I_87_ and minor patch around I_124_/L_125_. These residues are located at the entrance of ligand binding pocket of MD2 (S7 Fig). Together, with either LPS or neoseptin3, they form a large patch. In the absence of ligand, the patch is no longer contiguous and the binding affinity is reduced. An exception is F_126_ which has much larger contribution in the ligand-free tetramer than in the ligand-bound tetramers (Fig 3 and Table 3).

Not surprisingly, both LPS and neoseptin3 contribute to TLR4/MD2* and TLR4*/MD2 the most. Each of them contributes favorably to formation of these interfaces by -4.5 to -4.9 kcal/mol (Table 3) by forming direct contacts with TLR4. Furthermore, the interaction between LPS and TLR4 is driven favorably by the electrostatic and van der Waals forces that are compensated by unfavorable desolvation. In the neoseptin3 complex, each MD2 binds to two neoseptin3s. The contacts between neoseptin3 and TLR4 are also driven by favorable electrostatic and van der Waals forces with desolvation disfavors binding.

**Fig 4:**
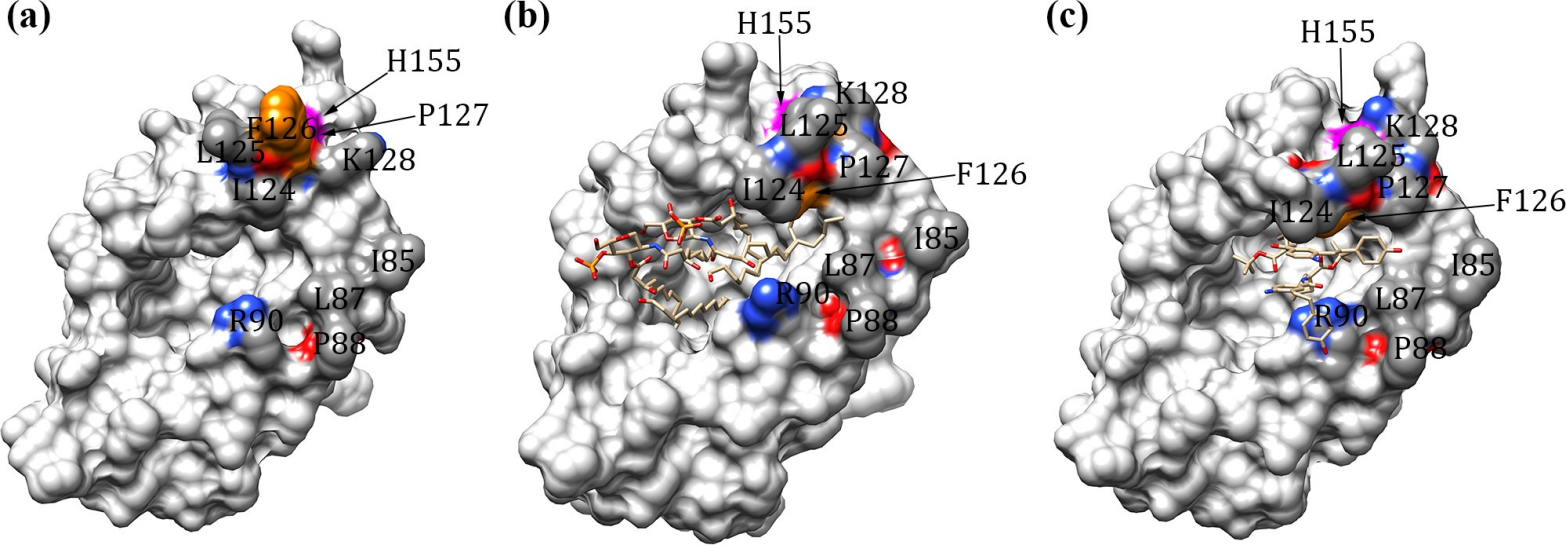
Illustration of key residues of MD2 in the TLR4/MD2* and TLR4*/MD2 interfaces. **a**) the ligand-free (TLR4-MD2)_2_ tetramer, **b)** the lipopolysaccharide (LPS)-bound (TLR4-MD2)_2_ tetramer, and **c)** the neoseptin3-bound (TLR4-MD2)_2_ tetramer complex. MD2 are shown in surface. LPS and neoseptin3 are shown in sticks. F_126_ and H_155_ are colored in orange and magenta, respectively. I_85_, L_87_, P_88_, R_90_, I_124_, L_125_, P_127_, K_128_ are colored by elements (red: oxygen; blue: nitrogen; gray: carbon) and the remaining residues of MD2 are colored in light gray. Displayed images were created with UCSF Chimera software [63].

The MD2 is a β-cup protein and its large central cavity is highly hydrophobic (Fig 4A). In the LPS complex, the central cavity is fully occupied by the hydrophobic tails of LPS with its phosphate head groups forming contact with TLR4 (Fig 4B). In neoseptin3 complex, this cavity is partially filled by two neoseptin3 molecules (Fig 4C). Thus, to enhance its potency, a potential avenue to explore is to design a compound that can fully fill the cavity with the exposed surface that complements TLR4 convex surface.

In their X-ray crystallography and mutagenesis studies, Kim et al. [45] found that F_126_ and H_155_ play important roles in tetramer formation. In our calculation, F_126_ in the ligand-free tetramer contributes -0.94 kcal/mol in TLR4/MD2* and -1.59 kcal/mol in TLR4*/MD2 interfaces and H_155_ contributes 0.34 kcal/mol in TLR4/MD2* and -0.05 kcal/mol in TLR4*/MD2 (Table 3). Thus, indeed, F_126_ plays important role in tetramer formation in the absence of ligands. However, with the ligands, contributions from F_126_ is significantly reduced. In the LPS-bound tetramer, F_126_ contributes only -0.38 kcal/mol in TLR4/MD2* and -0.48 kcal/mol in TLR4*/MD2 interface and H_155_ contributes only -0.07 kcal/mol in TLR4/MD2* and -0.08 kcal/mol in TLR4*/MD2 interface (Table 3), largely negligible. Similarly, in the neoseptin3-bound tetramer, contributions from F_126_ are -0.20 and -0.22 kcal/mol in TLR4/MD2* and TLR4*/MD2 interfaces, respectively and contributions from H_155_ are -0.08 and -0.13 kcal/mol in TLR4/MD2* and TLR4*/MD2 interfaces, respectively (Table 3). Thus, neither F_126_ nor H_155_ plays significant roles in formation of LPS-bound and neoseptin3-bound tetramer complexes. In fact, as shown in Fig 4, F_126_ is exposed without the ligands and can form contact with TLR4 across the subunits (Fig 4A), but is buried in both LPS-bound and neoseptin3-bound structures (Fig 3B and 3C). Therefore, our calculations are consistent with the X-ray structures. Instead, four neighboring residues, I_124_, L_125_, P_127_, and K_128_, are notably more exposed than F_126_ in the ligand-bound tetramers and have the potential to make significant contributions in tetramer formation. Indeed, in the ligand-free tetramer they contribute only -0.52, -1.05, -0.18, -1.14 kcal/mol, respectively, in the TLR4/MD2* interface and -1.55, -2.00, -0.25, -0.56 kcal/mol, respectively, in the TLR4*/MD2 (Table 3). In LPS-bound tetramer, their contributions increased substantially to -2.40, -2.60, -2.23, -1.10 kcal/mol, respectively, in the TLR4/MD2* interface, and -2.89, 2.98, -2.17, -1.06 kcal/mol, respectively, in the TLR4*/MD2 (Table 3).

On the other hand, the movement of F_126_ has been proposed as having significant functional implication. Indeed, in the ligand-free tetramer, F_126_ is exposed, can form direct contacts with TLR4 and facilitate tetramer formation whereas P_127_ is buried behind F_126_ (Fig 4). However, in LPS-bound and neoseptin3-bound tetramers, F_126_ forms close contacts with LPS and neoseptin3 and becomes completely buried. Instead, P_127_ becomes fully exposed, allowing formation of direct contacts with TLR4. Other notable structural changes include more exposure of I_124_ and L_125_. These three residues, I_124_, L_125_, P_127_, together contributes -7.2 to -8.1 kcal/mol free energy in LPS-bound complex and -6.7 to -8.4 kcal/mol in neoseptin3 complex in favor of tetramer formation (Table 3). Therefore, the hydrophobic I_124_-L_125_-P_127_ cluster is activated only when MD2 is bound to either LPS or neoseptin3.

In addition to the cluster formed by I_124_-L_125_-P_127_, interaction between MD2 and the convex surface of TLR4 is also facilitated by the cluster of I_85_-L_87_-P_88_-R_90_. In particular, I_85_ plays key roles in both the ligand-free and the ligand-bound complexes (Fig 3 and Table 3). The structure of the cluster is highly preserved between these two states and remains essentially the same (Fig 4). In the ligand-free tetramer, I_85_-L_87_-P_88_ together contribute -7.0 to -7.5 kcal/mol (Table 3). In LPS-bound and neoseptin3-bound tetramer, they together contribute -6.9 to -7.5 kcal/mol in LPS-bound complex and -7.0 to -7.3 kcal/mol in neoseptin3 complex (Table 3), both are significantly more than their contributions to the ligand-free complex of -1.7 to -3.8 kcal/mol. Given its similar roles in ligand-free and in ligand-bound tetramers, we deduce that the I_85_-L_87_-P_88_-R_90_ cluster facilitates tetramer formation, regardless whether MD2 is bound to a ligand or not.

It is important to note that the largest contribution to the binding free energy comes from the ligands themselves. In the LPS-bound complex, the LPS contributes to TLR4/MD2* and TLR4*/MD2 interfaces by -4.75 and -4.90 kcal/mol, respectively (Table 3). In neoseptin3 complex, its contributions are -4.86 and -4.47 kcal/mol (Table 3). These are more than the contributions by any of the protein residues. Thus, we propose that direct contacts between TLR4 and LPS, or neoseptin3 plays critical roles in TLR4-MD2 tetramer formation.

Moreover, to find the key residues of MD2 involved in ligand (either LPS or neoseptin3) interactions, we computed binding free energies of MD2/ligand and MD2*/ligand* interfaces in the LPS-bound and neoseptin3-bound (TLR4-MD2)_2_ tetramers (S10 Table and S10 Fig). F_121_ of MD2 is a key residue in both the LPS- and neoseptin3-bound tetramers. While R_90_ is a favorable key residue in the LPS-bound tetramer, it is unfavorable by an increase of 2.47 kcal/mol in the neoseptin3-bound tetramer. P_118_ is also unfavorable in both tetramers.

In the MM-PBSA and MM-GBSA free energy calculations, the entropic contribution can be estimated by either normal mode analysis or principle component analysis. Due to the large size of the systems, calculation of normal modes is rather challenging. Accurate calculation of the full principle components also requires extensive sampling to obtain a reliable estimation of the covariance matrix. For systems like (TLR4-MD2)_2_ tetramer, involving more than 23,000 atoms, such a matrix is extremely difficult to obtain. Thus, our discussions are qualitative and are based on the interaction energy and the solvation free energy. Nevertheless, we note that, without considering the reduction of entropy due to binding, the calculation can significantly over-estimate the binding free energies. For TLR4, the principle components of C_α_ atoms can capture large scale collective motion and can be used to illustrate the entropic changes for TLR4. To this end, entropy of the TLR4 monomer from the TLR4-MD2 is -6.69756 kcal/mol/K. In tetramers, the TLR4 monomer entropy is -6.72551, -6.84351, -6.87256 kcal/mol/K, for ligand-free, LPS-bound, and neoseptin3-bound tetramers, respectively. Indeed, formation of tetramer reduces the entropy of TLR4 monomers. At 300K, these entropy changes, relative to TLR4 in TLR4-MD2 heterodimer, would weaken the binding free energy by 8.4, 43.8, and 52.5 kcal/mol, for ligand-free, LPS-bound, and neoseptin3-bound tetramers, respectively. Further reduction is expected when the dynamics of MD2, relative movement of the subunits, and dynamics of the side chains are taken into consideration.

On the other hand, the TLR4 entropies calculated from the C_α_ atom collective motion, shows that TLR4 in LPS-bound and neoseptin3-bound tetramers is significantly more ordered than in the ligand-free complex. This is noteworthy since all three tetramers have essentially identical TLR4-MD2 heterodimer structures in which MD2 binds to the concave surface of TLR4 and have highly similar interaction patterns. Therefore, simple volumetric effect is insufficient to explain the difference. We propose that the reduced TLR4 monomer entropy in LPS-bound and neoseptin3-bound tetramers is due to the strong interactions between MD2 and TLR4 at the convex face around L_442_ of TLR4. Thus, presence of LPS or neoseptin3 not only stabilizes TLR4/MD2* and TLR4*/MD2 interfaces by direct contacts between the ligands and TLR4, it also stabilizes TLR4 monomer structure.

### Conclusion

TLR4-MD2 signaling plays a major physiological role in a broad spectrum of disorders including autoimmune disorders, asthma, cancer, and cardiovascular diseases. Therefore, TLR4 and MD2 have emerged as validated anti-inflammatory drug targets for acute inflammatory diseases such as septic shock and acute lung injury [12] and have also been proposed as anti-cancer targets. However, several anti-inflammatory clinical trials failed due to the nonspecific binding, loss of *in vivo* antagonistic activity, poor pharmacokinetic properties and low solubility. Future development in this field will encompass resolving these challenges, and discovering novel TLR4 or/and MD2 modulators as inhibitors or vaccine adjuvants with minimal off-target effects through developing therapeutic peptidomimetic small molecules and monoclonal antibody treatments [46].

Rational design of TLR4 and/or MD2 agonists and antagonists has become a hot research topic with the wealth of the structure biology information. The relatively recent availability of the X-ray crystallographic structures of the extracellular domain of TLR4-MD2 has opened new perspectives to identify the precise binding sites for structure-based drug design [12, 47]. Molecular modeling and computational chemistry techniques can benefit from these structures to unravel atomic details about the molecular recognition mechanism of the receptor itself and also about the ligand-receptor interactions of diverse modulators.

In this work, the role of TLR4-MD2 ligands in structural stabilization and conformational changes leading to tetramer formation was studied by extensive MD simulations and free energy calculations. Key residues in tetramer formation were identified that form two clusters on the MD2 surface located on both sides outside the LPS-binding cavity. These two clusters play critical roles in tetramer formation. Among them, the I_85_-L_87_-P_88_ hydrophobic cluster forms contacts with TLR4 in both ligand-free and ligand-bound tetramers. Thus, it may not be directly related to ligand-binding and activation. On the other hand, the I_124_-L_125_-P_127_ hydrophobic cluster forms contacts with TLR4 in the ligand-bound tetramers only. In particular, this cluster is formed after burial of F_126_ triggered by binding of LPS or neoseptin3. Thus, we proposed that this cluster plays key roles in TLR4-MD2 binding and activation by stabilizing (TLR4-MD2)_2_ tetramer which, in turn, helps to initiate a transmembrane conformational change that triggers adapter recruitment and signaling. In addition to these two hydrophobic clusters, direct contacts between TLR4 convex surface near L_442_ and LPS and neoseptin3 also contribute significantly to the binding between TLR4 and MD2-ligand. These new findings shed new lights to the mechanism of activation of TLR4-MD2 and can be utilized to design novel therapeutics that alter the dynamics of the TLR4-MD2 signaling receptor. Moreover, they can guide future experimental and mutagenesis studies.

## Methods

### TLR4 ectodomain models preparation

The ligand-bound and ligand-free crystal structures of the extracellular domain of mouse TLR4 and its co-receptor protein (MD2) were retrieved from the Protein Data Bank (PDB; accession codes are 5IJB, 5IJC, and 5IJD [35], for the mouse ligand-free, neoseptin3–bound, and LPS-bound structures, respectively). The ligand-bound (TLR4-MD2-LPS)_2_ and (TLR4-MD2-neoseptin3)_2_ structures are heterotetramers composed by two symmetrical dimeric copies of the TLR4–MD2 complexes and the ligands arranged in a symmetrical fashion (Fig 1). Under physiological concentration, the ligand-free TLR4-MD2 forms a heterodimer. However, under elevated concentration, TLR4-MD2 can also form a stable tetramer. Simulations on both ligand-free heterodimer TLR4-MD2 and ligand-free tetramer (TLR4-MD2)_2_ were performed for comparison.

The AMBER FF14SB [48] force field parameters were used to represent the proteins; small molecules (like LPS and neoseptin3) were represented by GAFF [49] force field and the charges were generated using RESP approach [50] with HF/6-31G* electrostatic potential after geometry optimization; polysaccharides were modeled using GLYCAM [51] force field. The prepared models and counter-ions (to neutralize the system) were solvated in a rectangular box filled with explicit TIP3P water [52]. With added counterions and water, the total system sizes were 223,350, 199,747, 209,125, and 117,233 atoms, for LPS-bound tetramer, neoseptin3-bound tetramer, ligand-free tetramer, and ligand-free TLR4-MD2 heterodimer, respectively.

### Molecular dynamics (MD) simulations

Four simulation trajectories were conducted for each system and each trajectory was simulated to 1.2 μs, making a total of 19.2 μs. All MD simulations were conducted using AMBER16 suite [40] on nVidia graphics processing units (GPUs) [53]. The particle mesh Ewald (PME) [54] approach was employed for long-range electrostatics using a 10 Å cutoff distance for van der Waals interactions. Steepest descent and conjugate gradient minimization were performed. Each system was prepared for the production simulation by following three-stage equilibration process. During the first stage, velocities were randomly assigned at 10K and the systems were gradually heated to 300K in 10 steps (approximately 30K per step) in 50 ps under a constant volume (NVT) ensemble with a time step of 1.0 fs. During the second stage, the systems were further equilibrated for 500 ps with 1.0 fs time step in constant pressure ensemble (NPT) using the Berendsen barostat [55] under an isotropic pressure of 1.0 bar. In the third equilibration stage, the systems were further equilibrated by 200.0 ns MD simulations with a time step of 2.0 fs in constant pressure ensemble using the Monte-Carlo barostat at 1.0 bar and the temperature was maintained at 300 K. The production MD simulations were performed for 1.0 μs with simulation protocols identical to those used in the third equilibration stage. The SHAKE algorithm [56] was applied to constrain bond lengths to all hydrogen atoms to allow a 2.0 fs time step. Temperature was controlled by the Langevin thermostat method [57]. Four simulations were performed for each complex, differing only on the initial distribution of velocities, to allow scrutiny of the reproducibility of the results. Analyses on the trajectories were performed using CPPTRAJ [58] program and in-house scripts.

### Free energy calculations

The end-state binding free energies between the TLR4/MD2, TLR4/MD2* (and their symmetry-related images TLR4*/MD2* and TLR4*/MD2, respectively), TLR4/TLR4*, and (TLR4-MD2)/(TLR4*-MD2*) interfaces (Fig 1) were estimated with both MM-GBSA and MM-PBSA methods using the parallelized python script *MMPBSA*.*py*.*MPI* [59] in Amber 16. A total of 4000 frames for each system were used in the free energy calculations (1000 frames from the last 1.0 μs of each MD trajectory with an interval of 1.0 ns). In these methods, the binding free energy (Δ*G*) can be represented as:

$$\Delta G=\Delta E_{MM}+{\Delta G}_{sol}-T\Delta S$$

where Δ*E*_*mm*_ and *T*Δ*S* are the molecular mechanics free energy and the conformational entropy effect in the gas phase, respectively, and Δ*G*_*sol*_ is the solvation free energy. In our calculation, the entropy portion of the free energy was not considered due to the challenge to accurately calculate the entropy for such a large system and the fact we intend to provide a qualitative understanding of the system and focus on the difference between the systems. Δ*E*_*mm*_ can be further divided into two parts:

$${\Delta E}_{MM}={\Delta E}_{ele}+{\Delta E}_{vdw}$$

in which Δ*E*_*ele*_ and Δ*E*_*vdw*_ represent the contributions from the electrostatic and van der Waals interactions, respectively calculated based on the force field used in the MD simulations. The solvation free energy (Δ*G*_*sol*_) can be expressed as:

$${\Delta G}_{sol}={\Delta G}_{pol}+{\Delta G}_{nonpol}$$

where Δ*G*_*pol*_ and Δ*G*_*nonpol*_ are the polar and non-polar contributions to the solvation free energy, respectively. Δ*G*_*pol*_ is calculated using the Poisson–Boltzmann (PB) [60, 61] or modified Generalized Born (GB) model developed by Onufriev et al. [62]. Δ*G*_*nonpol*_ was estimated by:

$${\Delta G}_{SA}=\gamma SASA+b$$

where the symbol *SASA* denotes the solvent accessible surface area which was computed using a probe radius of 1.4 Å. In this work, the values for *γ* and *b* were set to 0.005 kcal/(mol.Å^2^) and 0.0 kcal/mol, respectively. The per-residue based energy decomposition was performed to identify the key residues in each interface.

## Supporting information

S1 Fig The per residue energy contribution spectrums of TLR4 and MD2 in the TLR4/MD2 interface. **a)** the ligand-free TLR4-MD2 hetero dimer, **b)** the (TLR4-MD2)_2_ tetramer, **c)** the lipopolysaccharide (LPS)-bound (TLR4-MD2)_2_ tetramer, and **d)** the neoseptin3-bound (TLR4-MD2)_2_ tetramer complex. The favorable key residues (lower than -2 kcal/mol) and unfavorable residues (greater than 2 kcal/mol) are shown in black and blue, respectively.
(TIF)

S2 Fig Illustration of the key residues in the TLR4/MD2 interface. **a)** the ligand-free TLR4-MD2 heterodimer, **b)** the (TLR4-MD2)_2_ tetramer, **c)** the lipopolysaccharide (LPS)-bound (TLR4-MD2)_2_ tetramer, and **d)** the neoseptin3-bound (TLR4-MD2)_2_ tetramer complex. The favorable and unfavorable residues are colored in red and blue, respectively and the ligands (LPS or neoseptin3) are colored in yellow. The TLR4 and MD2 monomers are rotated for the best view.
(TIF)

S3 Fig The per residue energy contribution spectrums of TLR4* and MD2* in the TLR4*/MD2* interface. **a)** the ligand-free (TLR4-MD2)_2_ tetramer, **b)** the lipopolysaccharide (LPS)-bound (TLR4-MD2)_2_ tetramer, and **c)** the neoseptin3-bound (TLR4-MD2)_2_ tetramer complex. The favorable key residues (lower than -2 kcal/mol) and unfavorable residues (greater than 2 kcal/mol) are shown in black and blue, respectively.
(TIF)

S4 Fig Illustration of the key residues in the TLR4*/MD2* interface. **a)** the ligand-free (TLR4-MD2)2 tetramer, **b)** the lipopolysaccharide (LPS)-bound (TLR4-MD2)_2_ tetramer, and **c)** the neoseptin3-bound (TLR4-MD2)_2_ tetramer complex. The favorable and unfavorable residues are colored in red and blue, respectively and the ligands (LPS or neoseptin3) are colored in yellow. The TLR4* and MD*2 monomers are rotated for the best view.
(TIF)

S5 Fig The per residue energy contribution spectrums of TLR4 and TLR4* in the TLR4/TLR4* interface. **a)** the ligand-free (TLR4-MD2)_2_ tetramer, **b)** the lipopolysaccharide (LPS)-bound (TLR4-MD2)_2_ tetramer, and **c)** the neoseptin3-bound (TLR4-MD2)_2_ tetramer complex. The favorable key residues (lower than -2 kcal/mol) and unfavorable residues (greater than 2 kcal/mol) are shown in black and blue, respectively.
(TIF)

S6 Fig Illustration of the key residues in the TLR4/TLR4* interface. **a)** the ligand-free (TLR4-MD2)_2_ tetramer, **b)** the lipopolysaccharide (LPS)-bound (TLR4-MD2)_2_ tetramer, and **c)** the neoseptin3-bound (TLR4-MD2)_2_ tetramer complex. The favorable and unfavorable residues are colored in red and blue, respectively. The TLR4* monomer has been shown in a more transparent representation.
(TIF)

S7 Fig Illustration of the key residues in the TLR4/MD2* interface. **a)** the ligand-free (TLR4-MD2)_2_ tetramer, **b)** the lipopolysaccharide (LPS)-bound (TLR4-MD2)_2_ tetramer, and **c)** the neoseptin3-bound (TLR4-MD2)_2_ tetramer complex. The favorable and unfavorable residues are colored in red and blue, respectively and the ligands (LPS or neoseptin3) are colored in yellow. The TLR4 and MD*2 monomers are rotated for the best view.
(TIF)

S8 Fig The per residue energy contribution spectrums of TLR4* and MD2 in the TLR4*/MD2 interface. **a)** the ligand-free (TLR4-MD2)_2_ tetramer, **b)** the lipopolysaccharide (LPS)-bound (TLR4-MD2)_2_ tetramer, and **c)** the neoseptin3-bound (TLR4-MD2)_2_ tetramer complex. The favorable key residues (lower than -2 kcal/mol) and unfavorable residues (greater than 2 kcal/mol) are shown in black and blue, respectively.
(TIF)

S9 Fig Illustration of the key residues in the TLR4*/MD2 interface. **a)** the ligand-free (TLR4-MD2)_2_ tetramer, **b)** the lipopolysaccharide (LPS)-bound (TLR4-MD2)_2_ tetramer, and **c)** the neoseptin3-bound (TLR4-MD2)_2_ tetramer complex. The favorable and unfavorable residues are colored in red and blue, respectively and the ligands (LPS or neoseptin3) are colored in yellow. The TLR4* and MD2 monomers are rotated for the best view.
(TIF)

S10 Fig The per residue energy contribution spectrums of MD2, MD2* and ligands (LPS, neoseptin3) in the MD2/ligand or MD2*/ligand interface of a and b) the lipopolysaccharide (LPS)-bound (TLR4-MD2)2 tetramer complex, c and d) the neoseptin3-bound (TLR4-MD2)2 tetramer complex. The favorable key residues (lower than -2 and -1 kcal/mol) and unfavorable residues (greater than 1 kcal/mol) are shown in black and blue, respectively. The Illustration of these residues are shown next to each spectrum. The favorable and unfavorable residues are colored in red and blue, respectively and the ligands (LPS or neoseptin3) are colored in yellow.
(TIF)

S1 Table The C_α_ root mean-square deviation (RMSD) of ligand-free TLR4-MD2 heterodimer, (TLR4-MD2)_2_ tetramer, lipopolysaccharide (LPS)-bound (TLR4-MD2)_2_ tetramer, and neoseptin3-bound (TLR4-MD2)_2_ tetramer complexes are averaged over the last 1.0 μs of each trajectory. Trajectories in each complex are identified with a number from 1 to 4 (#) and their average is denoted by ‘1–4’ as shaded in light grey. The values in parenthesis are standard deviation.
(PDF)

S2 Table The binding free energies (Δ*G*) in kcal/mol computed by both molecular mechanics generalized Born surface area (MM-GBSA) and molecular mechanics Poisson-Boltzmann surface area (MM-PBSA) methods at TLR4/MD2 interface. The free energies in each trajectory of the ligand-free TLR4-MD2 heterodimer, (TLR4-MD2)_2_ tetramer, lipopolysaccharide (LPS)-bound (TLR4-MD2)_2_ tetramer, and neoseptin3-bound (TLR4-MD2)_2_ tetramer complexes are averaged over the 1000 frames from the last 1.0 μs of that trajectory with an interval of 1.0 ns. Trajectories in each complex are identified with a number from 1 to 4 (#) and their average is denoted by ‘1–4’ as shaded in light grey. A negative value is a favorable free energy, while a positive value is an unfavorable. The values in parenthesis are standard deviation. Δ*EMM* is molecular mechanics free energy which is divided into Δ*Eele* and Δ*Evdw* representing the contributions from the electrostatic and van der Waals interactions, respectively. Δ*Gsol* is solvation free energy expressed by Δ*Gpol* and Δ*Gpol*, the polar and non-polar contributions, respectively.
(PDF)

S3 Table The binding free energies (Δ*G*) in kcal/mol computed by both molecular mechanics generalized Born surface area (MM-GBSA) and molecular mechanics Poisson-Boltzmann surface area (MM-PBSA) methods at TLR4*/MD2* interface. (PDF)

S4 Table The binding free energies (Δ*G*) in kcal/mol computed by both molecular mechanics generalized Born surface area (MM-GBSA) and molecular mechanics Poisson-Boltzmann surface area (MM-PBSA) methods at TLR4/TLR4* interface. (PDF)

S5 Table The binding free energies (Δ*G*) in kcal/mol computed by both molecular mechanics generalized Born surface area (MM-GBSA) and molecular mechanics Poisson-Boltzmann surface area (MM-PBSA) methods at TLR4/MD2* interface. (PDF)

S6 Table The binding free energies (Δ*G*) in kcal/mol computed by both molecular mechanics generalized Born surface area (MM-GBSA) and molecular mechanics Poisson-Boltzmann surface area (MM-PBSA) methods at TLR4*/MD2 interface. (PDF)

S7 Table The binding free energies (Δ*G*) in kcal/mol at (TLR4-MD2)/(TLR4*-MD2*) interface computed by both molecular mechanics generalized Born surface area (MM-GBSA) and molecular mechanics Poisson-Boltzmann surface area (MM-PBSA) methods. The free energies in each trajectory of the ligand-free (TLR4-MD2)_2_ tetramer, lipopolysaccharide (LPS)-bound (TLR4-MD2)_2_ tetramer, and neoseptin3-bound (TLR4-MD2)_2_ tetramer complexes are averaged over the 1000 frames from the last 1.0 μs of that trajectory with an interval of 1.0 ns. Trajectories in each complex are identified with a number from 1 to 4 (#) and their average is denoted by ‘1–4’ as shaded in light grey. A negative value is a favorable free energy, while a positive value is an unfavorable. The values in parenthesis are standard deviation. Δ*EMM* is molecular mechanics free energy which is divided into Δ*Eele* and Δ*Evdw* representing the contributions from the electrostatic and van der Waals interactions, respectively. Δ*Gsol* is solvation free energy expressed by Δ*Gpol* and Δ*Gnonpol*, the polar and non-polar contributions, respectively.
(PDF)

S8 Table The key residues of TLR4 and MD2 identified by per-residue free energy decomposition (kcal/mol) in the TLR4/MD2 and TLR4*/MD2* interfaces of ligand-free TLR4-MD2 heterodimer, (TLR4-MD2)2 tetramer, lipopolysaccharide (LPS)-bound (TLR4-MD2)2 tetramer, and neoseptin3-bound (TLR4-MD2)2 tetramer complexes. The values are averaged over the 1000 frames of the combined 4 trajectories of each system. A negative value is a favorable free energy, while a positive value is an unfavorable. The shaded entries are those that have unfavorable free energy contribution of 2.0 kcal/mol or greater.
(PDF)

S9 Table The key residues of TLR4 and TLR4* identified by per-residue free energy decomposition (kcal/mol) in the TLR4/TLR4* interface of ligand-free (TLR4-MD2)2 tetramer, lipopolysaccharide (LPS)-bound (TLR4-MD2)2 tetramer, and neoseptin3-bound (TLR4-MD2)2 tetramer complexes. The values are averaged over the 1000 frames of the combined 4 trajectories of each system. A negative value is a favorable free energy, while a positive value is an unfavorable. The shaded entries are those that have unfavorable free energy contribution of 2.0 kcal/mol or greater.
(PDF)

S10 Table The binding free energies (Δ*G*) in kcal/mol between either MD2 or MD2* monomer and the ligands (either LPS or neoseptin3) at either the MD2/ligand or MD2*/ligand interface computed by both molecular mechanics generalized Born surface area (MM-GBSA) and molecular mechanics Poisson-Boltzmann surface area (MM-PBSA) methods. The free energies in each of the lipopolysaccharide (LPS)-bound (TLR4-MD2)_2_ tetramer and neoseptin3-bound (TLR4-MD2)_2_ tetramer complexes are averaged over the 1000 frames of the combined 4 trajectories. A negative value is a favorable free energy, while a positive value is an unfavorable. The values in parenthesis are standard deviation. Δ*E*_*MM*_ is molecular mechanics free energy which is divided into Δ*E*_*ele*_ and Δ*E*_*vdw*_ representing the contributions from the electrostatic and van der Waals interactions, respectively. Δ*G*_*sol*_ is solvation free energy expressed by Δ*G*_*pol*_ and Δ*G*_*nonpol*_, the polar and non-polar contributions, respectively.
(PDF)

## References

[1] Medzhitov R. Toll-like receptors and innate immunity. Nat Rev Immunol. 2001;1:135–145. 10.1038/35100529 11905821

[2] Netea MG, Wijmenga C, O'neill LA. Genetic variation in Toll-like receptors and disease susceptibility. Nat Immunol. 2012;13:535–542. 10.1038/ni.2284 22610250

[3] De Nardo D. Toll-like receptors: Activation, signalling and transcriptional modulation. Cytokine. 2015;74:181–189. 10.1016/j.cyto.2015.02.025 25846205

[4] Gay NJ, Gangloff M, Weber AN. Toll-like receptors as molecular switches. Nat Rev Immunol. 2006;6:693–698. 10.1038/nri1916 16917510

[5] Gay NJ, Symmons MF, Gangloff M, Bryant CE. Assembly and localization of Toll-like receptor signalling complexes. Nat Rev Immunol. 2014;14:546–558. 10.1038/nri3713 25060580

[6] Miguel RN, Wong J, Westoll JF, Brooks HJ, O'Neill LA, Gay NJ, et al A dimer of the Toll-like receptor 4 cytoplasmic domain provides a specific scaffold for the recruitment of signalling adaptor proteins. PLoS One. 2007;2:e788 10.1371/journal.pone.0000788 17726518 PMC1945083

[7] Poltorak A, He X, Smirnova I, Liu M-Y, Van Huffel C, Du X, et al Defective LPS signaling in C3H/HeJ and C57BL/10ScCr mice: mutations in Tlr4 gene. Science. 1998;282:2085–2088. 10.1126/science.282.5396.2085 9851930

[8] Hoshino K, Takeuchi O, Kawai T, Sanjo H, Ogawa T, Takeda Y, et al Cutting edge: Toll-like receptor 4 (TLR4)-deficient mice are hyporesponsive to lipopolysaccharide: evidence for TLR4 as the Lps gene product. J Immunol. 1999;162:3749–3752. 10201887

[9] Shimazu R, Akashi S, Ogata H, Nagai Y, Fukudome K, Miyake K, et al MD-2, a molecule that confers lipopolysaccharide responsiveness on Toll-like receptor 4. J Exp Med. 1999;189:1777–1782. 10.1084/jem.189.11.1777 10359581 PMC2193086

[10] Cook DN, Pisetsky DS, Schwartz DA. Toll-like receptors in the pathogenesis of human disease. Nat Immunol. 2004;5:975–979. 10.1038/ni1116 15454920

[11] Roger T, Froidevaux C, Le Roy D, Reymond MK, Chanson A-L, Mauri D, et al Protection from lethal gram-negative bacterial sepsis by targeting Toll-like receptor 4. Proc Natl Acad Sci USA. 2009;106:2348–2352. 10.1073/pnas.0808146106 19181857 PMC2650125

[12] Chen L, Fu W, Zheng L, Wang Y, Liang G. Recent progress in the discovery of myeloid differentiation 2 (MD2) modulators for inflammatory diseases. Drug Discov Today. 2018;23:1187–1202. 10.1016/j.drudis.2018.01.015 29330126

[13] Xie L, Jiang F-C, Zhang L-M, He W-T, Liu J-H, Li M-Q, et al Targeting of MyD88 homodimerization by novel synthetic inhibitor TJ-M2010-5 in preventing colitis-associated colorectal cancer. J Natl Cancer Inst. 2016;108. 10.1093/jnci/djv364 26712311

[14] Rakoff-Nahoum S, Medzhitov R. Toll-like receptors and cancer. Nat Rev Cancer. 2009;9:57 10.1038/nrc2541 19052556

[15] Kelly MG, Alvero AB, Chen R, Silasi D-A, Abrahams VM, Chan S, et al TLR-4 signaling promotes tumor growth and paclitaxel chemoresistance in ovarian cancer. Cancer Res. 2006;66:3859–3868. 10.1158/0008-5472.CAN-05-3948 16585214

[16] Bhatelia K, Singh K, Singh R. TLRs: linking inflammation and breast cancer. Cell Signal. 2014;26:2350–2357. 10.1016/j.cellsig.2014.07.035 25093807

[17] Frantz S, Ertl G, Bauersachs J. Mechanisms of disease: Toll-like receptors in cardiovascular disease. Nat Clin Pract Cardiovasc Med. 2007;4:444–454. 10.1038/ncpcardio0938 17653117

[18] Satoh M, Ishikawa Y, Minami Y, Takahashi Y, Nakamura M. Role of Toll like receptor signaling pathway in ischemic coronary artery disease. Front Biosci. 2007;13:6708–6715. 10.2741/3183 18508689

[19] Fukata M, Abreu M. TLR4 signalling in the intestine in health and disease. Biochem Soc Trans. 2007;35:1473–1478. 10.1042/BST0351473 18031248

[20] Pine SO, McElrath MJ, Bochud P-Y. Polymorphisms in TLR4 and TLR9 influence viral load in a sero-incident cohort of HIV-1-infected individuals. AIDS. 2009;23:2387–2395. 10.1097/QAD.0b013e328330b489 19855253 PMC2919356

[21] Balistreri C, Grimaldi M, Chiappelli M, Licastro F, Castiglia L, Listì F, et al Association between the polymorphisms of TLR4 and CD14 genes and Alzheimer's disease. Curr Pharm Des. 2008;14:2672–2677. 10.2174/138161208786264089 19006850

[22] van den Berg WB, van Lent PL, Joosten LA, Abdollahi-Roodsaz S, Koenders MI. Amplifying elements of arthritis and joint destruction. Ann Rheum Dis. 2007;66:iii45–iii48. 10.1136/ard.2007.079830 17934095 PMC2095284

[23] Goh FG, Midwood KS. Intrinsic danger: activation of Toll-like receptors in rheumatoid arthritis. Rheumatology. 2011;51:7–23. 10.1093/rheumatology/ker257 21984766

[24] Anders H-J, Banas B, Schlöndorff D. Signaling danger: toll-like receptors and their potential roles in kidney disease. J Am Soc Nephrol. 2004;15:854–867. 10.1097/01.asn.0000121781.89599.16 15034087

[25] Kim JK. Fat uses a TOLL-road to connect inflammation and diabetes. Cell Metab. 2006;4:417–419. 10.1016/j.cmet.2006.11.008 17141623

[26] O'Neill LA, Bryant CE, Doyle SL. Therapeutic targeting of Toll-like receptors for infectious and inflammatory diseases and cancer. Pharmacol Rev. 2009;61:177–197. 10.1124/pr.109.001073 19474110 PMC2846156

[27] Savva A, Roger T. Targeting toll-like receptors: promising therapeutic strategies for the management of sepsis-associated pathology and infectious diseases. Front Immunol. 2013;4:387 10.3389/fimmu.2013.00387 24302927 PMC3831162

[28] Peri F, Calabrese V. Toll-like receptor 4 (TLR4) modulation by synthetic and natural compounds: an update: miniperspective. J Med Chem. 2013;57:3612–3622. 10.1021/jm401006s 24188011 PMC4040398

[29] Kanzler H, Barrat FJ, Hessel EM, Coffman RL. Therapeutic targeting of innate immunity with Toll-like receptor agonists and antagonists. Nat Med. 2007;13:552–559. 10.1038/nm1589 17479101

[30] Rice TW, Wheeler AP, Bernard GR, Vincent J-L, Angus DC, Aikawa N, et al A randomized, double-blind, placebo-controlled trial of TAK-242 for the treatment of severe sepsis. Crit Care Med. 2010;38:1685–1694. 10.1097/CCM.0b013e3181e7c5c9 20562702

[31] Rossignol DP, Lynn M. Antagonism of in vivo and ex vivo response to endotoxin by E5564, a synthetic lipid A analogue. J Endotoxin Res. 2002;8:483–488. 10.1179/096805102125001127 12697095

[32] Barochia A, Solomon S, Cui X, Natanson C, Eichacker PQ. Eritoran tetrasodium (E5564) treatment for sepsis: review of preclinical and clinical studies. Expert Opin Drug Metab Toxicol. 2011;7:479–494. 10.1517/17425255.2011.558190 21323610 PMC3065179

[33] Opal SM, Laterre P-F, Francois B, LaRosa SP, Angus DC, Mira J-P, et al Effect of eritoran, an antagonist of MD2-TLR4, on mortality in patients with severe sepsis: the ACCESS randomized trial. JAMA. 2013;309:1154–1162. 10.1001/jama.2013.2194 23512062

[34] Morin MD, Wang Y, Jones BT, Su LJ, Surakattula M, Berger M, et al Discovery and Structure-Activity Relationships of the Neoseptins: A New Class of Toll-like Receptor-4 (TLR4) Agonists. J Med Chem. 2016;59:4812–4830. 10.1021/acs.jmedchem.6b00177 WOS:000376840600031. 27050713 PMC4882283

[35] Wang Y, Su L, Morin MD, Jones BT, Whitby LR, Surakattula MMRP, et al TLR4/MD-2 activation by a synthetic agonist with no similarity to LPS. Proc Natl Acad Sci USA. 2016;113:E884–E893. 10.1073/pnas.1525639113 26831104 PMC4763747

[36] Huber RG, Berglund NA, Kargas V, Marzinek JK, Holdbrook DA, Khalid S, et al A Thermodynamic funnel drives bacterial lipopolysaccharide transfer in the TLR4 pathway. Structure. 2018;26:1151–1161. e1154. 10.1016/j.str.2018.04.007 29779787

[37] Ryu J-K, Kim SJ, Rah S-H, Kang JI, Jung HE, Lee D, et al Reconstruction of LPS transfer cascade reveals structural determinants within LBP, CD14, and TLR4-MD2 for efficient LPS recognition and transfer. Immunity. 2017;46:38–50. 10.1016/j.immuni.2016.11.007 27986454

[38] Akashi S, Nagai Y, Ogata H, Oikawa M, Fukase K, Kusumoto S, et al Human MD-2 confers on mouse Toll-like receptor 4 species-specific lipopolysaccharide recognition. Int Immunol. 2001;13:1595–1599. 10.1093/intimm/13.12.1595 11717200

[39] Park BS, Song DH, Kim HM, Choi B-S, Lee H, Lee J-O. The structural basis of lipopolysaccharide recognition by the TLR4–MD-2 complex. Nature. 2009;458:1191–1195. 10.1038/nature07830 19252480

[40] Case DA, Cheatham TE, Darden T, Gohlke H, Luo R, Merz KM, et al The Amber biomolecular simulation programs. J Comput Chem. 2005;26:1668–1688. 10.1002/jcc.20290 16200636 PMC1989667

[41] Pearlman DA, Case DA, Caldwell JW, Ross WS, Cheatham TE, DeBolt S, et al AMBER, a package of computer programs for applying molecular mechanics, normal mode analysis, molecular dynamics and free energy calculations to simulate the structural and energetic properties of molecules. Comput Phys Commun. 1995;91:1–41.

[42] de Aguiar C, Costa MG, Verli H. Dynamics on human Toll‐like receptor 4 complexation to MD‐2: The coreceptor stabilizing function. Proteins. 2015;83:373–382. 10.1002/prot.24739 25488602

[43] Anwar MA, Choi S. Structure-Activity Relationship in TLR4 Mutations: Atomistic Molecular Dynamics Simulations and Residue Interaction Network Analysis. Sci Rep. 2017;7:43807 10.1038/srep43807 28272553 PMC5341570

[44] Paramo T, Piggot TJ, Bryant CE, Bond PJ. The Structural Basis for Endotoxin-induced Allosteric Regulation of the Toll-like Receptor 4 (TLR4) Innate Immune Receptor. J Biol Chem 12 20;288(51): 10.1074/jbc.M113.501957 Epub 2013 Oct 30. 2013;288:36215–36225. 24178299 PMC3868736

[45] Kim HM, Park BS, Kim J-I, Kim SE, Lee J, Oh SC, et al Crystal structure of the TLR4-MD-2 complex with bound endotoxin antagonist Eritoran. Cell. 2007;130:906–917. 10.1016/j.cell.2007.08.002 17803912

[46] Daubeuf B, Mathison J, Spiller S, Hugues S, Herren S, Ferlin W, et al TLR4/MD-2 monoclonal antibody therapy affords protection in experimental models of septic shock. J Immunol. 2007;179:6107–6114. 10.4049/jimmunol.179.9.6107 17947685

[47] Billod J-M, Lacetera A, Guzmán-Caldentey J, Martín-Santamaría S. Computational approaches to toll-like receptor 4 modulation. Molecules. 2016;21:994. 10.3390/molecules21080994 PMC6274477 27483231

[48] Maier JA, Martinez C, Kasavajhala K, Wickstrom L, Hauser KE, Simmerling C. ff14SB: improving the accuracy of protein side chain and backbone parameters from ff99SB. J Chem Theory Comput. 2015;11:3696–3713. 10.1021/acs.jctc.5b00255 26574453 PMC4821407

[49] Wang J, Wolf RM, Caldwell JW, Kollman PA, Case DA. Development and testing of a general amber force field. J Comput Chem. 2004;25:1157–1174. 10.1002/jcc.20035 15116359

[50] Bayly CI, Cieplak P, Cornell WD, Kollman PA. A Well-Behaved Electrostatic Potential Based Method Using Charge Restraints for Deriving Atomic Charges—the Resp Model. J Phys Chem. 1993;97:10269–10280.

[51] Kirschner KN, Yongye AB, Tschampel SM, González‐Outeiriño J, Daniels CR, Foley BL, et al GLYCAM06: a generalizable biomolecular force field. Carbohydrates. J Comput Chem. 2008;29:622–655. 10.1002/jcc.20820 17849372 PMC4423547

[52] Jorgensen WL, Chandrasekhar J, Madura JD, Impey RW, Klein ML. Comparison of simple potential functions for simulating liquid water. J Chem Phys. 1983;79:926–935.

[53] Le Grand S, Götz AW, Walker RC. SPFP: Speed without compromise—A mixed precision model for GPU accelerated molecular dynamics simulations. Comput Phys Commun. 2013;184:374–380.

[54] Darden T, York D, Pedersen L. Particle mesh Ewald: An N⋅ log (N) method for Ewald sums in large systems. J Chem Phys. 1993;98:10089–10092.

[55] Berendsen HJ, Postma Jv, van Gunsteren WF, DiNola A, Haak J. Molecular dynamics with coupling to an external bath. J Chem Phys. 1984;81:3684–3690.

[56] Ryckaert J-P, Ciccotti G, Berendsen HJ. Numerical integration of the cartesian equations of motion of a system with constraints: molecular dynamics of n-alkanes. J Comput Phys. 1977;23:327–341.

[57] Pastor RW, Brooks BR, Szabo A. An analysis of the accuracy of Langevin and molecular dynamics algorithms. Mol Phys. 1988;65:1409–1419.

[58] Roe DR, Cheatham TE III. PTRAJ and CPPTRAJ: software for processing and analysis of molecular dynamics trajectory data. J Chem Theory Comput. 2013;9:3084–3095. 10.1021/ct400341p 26583988

[59] Miller BR III, McGee TD Jr, Swails JM, Homeyer N, Gohlke H, Roitberg AE. MMPBSA. py: an efficient program for end-state free energy calculations. J Chem Theory Comput. 2012;8:3314–3321. 10.1021/ct300418h 26605738

[60] Honig B, Nicholls A. Classical electrostatics in biology and chemistry. Science. 1995;268:1144–1149. 10.1126/science.7761829 7761829

[61] Tan C, Tan Y-H, Luo R. Implicit nonpolar solvent models. J Phys Chem B. 2007;111:12263–12274. 10.1021/jp073399n 17918880

[62] Onufriev A, Bashford D, Case DA. Exploring protein native states and large‐scale conformational changes with a modified generalized born model. Proteins. 2004;55:383–394. 10.1002/prot.20033 15048829

[63] Pettersen EF, Goddard TD, Huang CC, Couch GS, Greenblatt DM, Meng EC, et al UCSF Chimera—a visualization system for exploratory research and analysis. J Comput Chem. 2004;25:1605–1612. 10.1002/jcc.20084 15264254

