# Supplementary figures and images for: Key residues in TLR4-MD2 tetramer formation identified by free energy simulations
Source: PLoS Comput Biol. 2019 Oct 14;15(10):e1007228. doi: 10.1371/journal.pcbi.1007228 (PMC6812856; doi:10.1371/journal.pcbi.1007228)

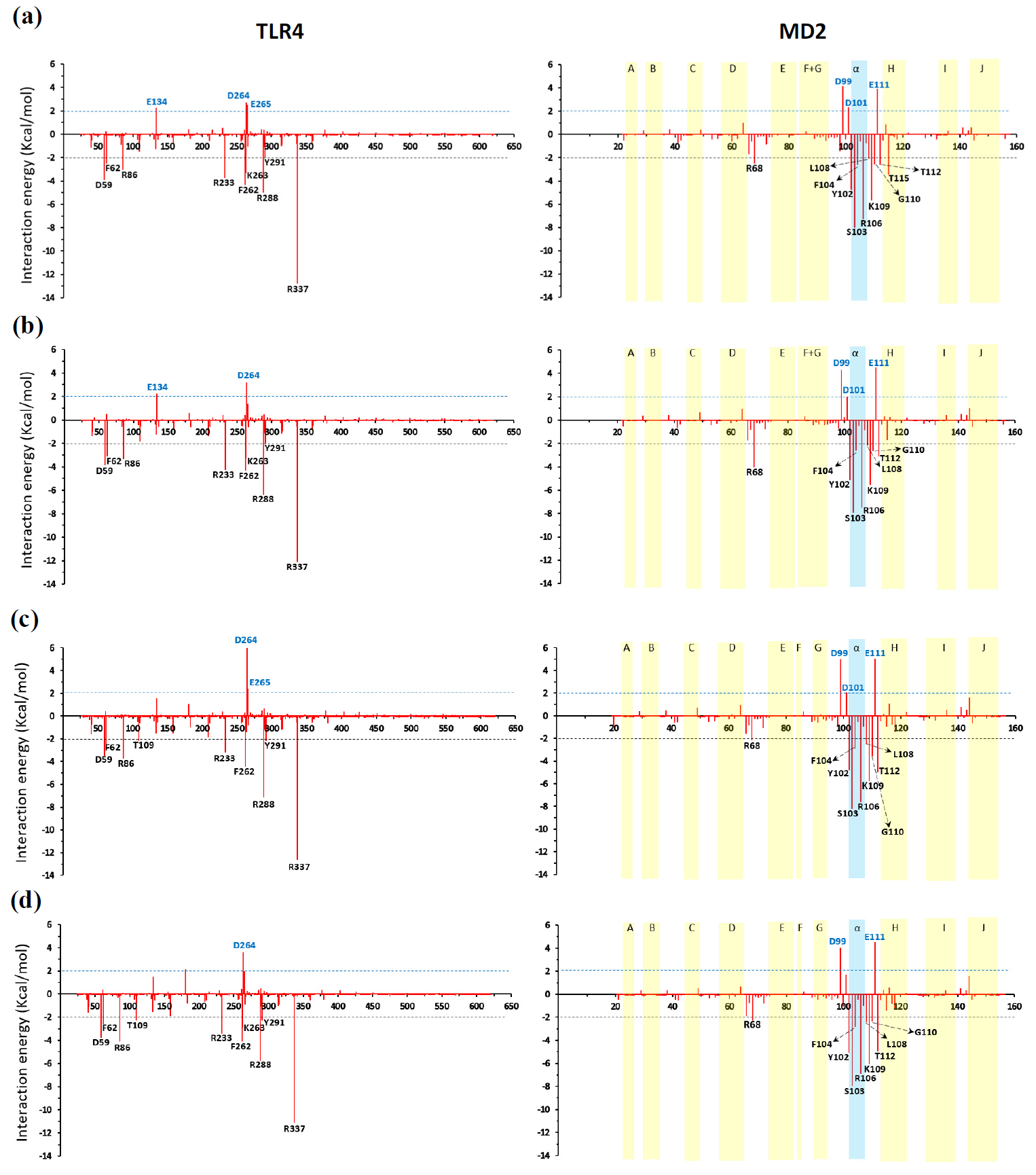

Supplement: S1 Fig — a) the ligand-free TLR4-MD2 hetero dimer, b) the (TLR4-MD2)2 tetramer, c) the lipopolysaccharide (LPS)-bound (TLR4-MD2)2 tetramer, and d) the neoseptin3-bound (TLR4-MD2)2 tetramer complex. The favorable key residues (lower than -2 kcal/mol) and unfavorable residues (greater than 2 kcal/mol) are shown in black and blue, respectively. (TIF) [file pcbi.1007228.s001.tif]

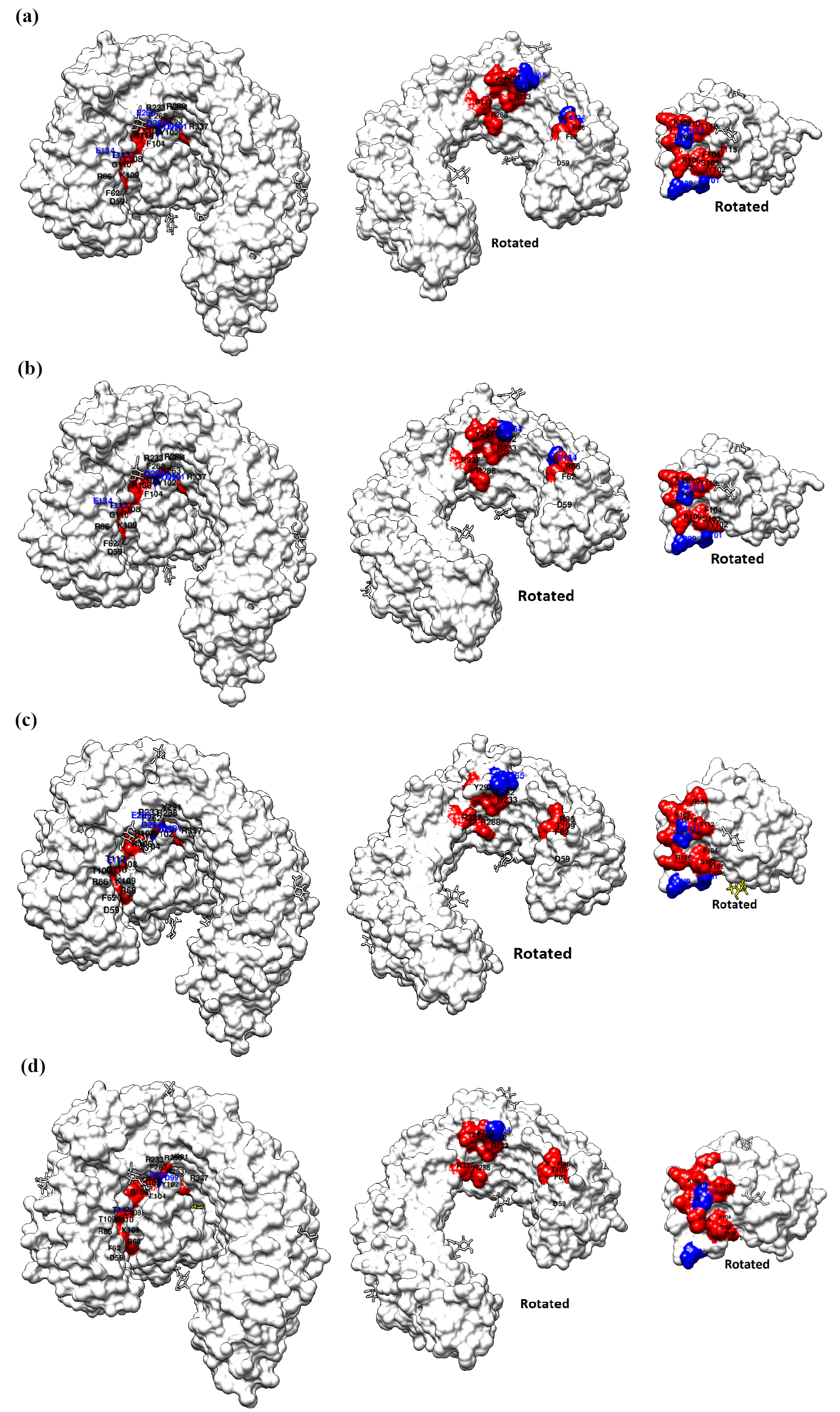

Supplement: S2 Fig — a) the ligand-free TLR4-MD2 heterodimer, b) the (TLR4-MD2)2 tetramer, c) the lipopolysaccharide (LPS)-bound (TLR4-MD2)2 tetramer, and d) the neoseptin3-bound (TLR4-MD2)2 tetramer complex. The favorable and unfavorable residues are colored in red and blue, respectively and the ligands (LPS or neoseptin3) are colored in yellow. The TLR4 and MD2 monomers are rotated for the best view. (TIF) [file pcbi.1007228.s002.tif]

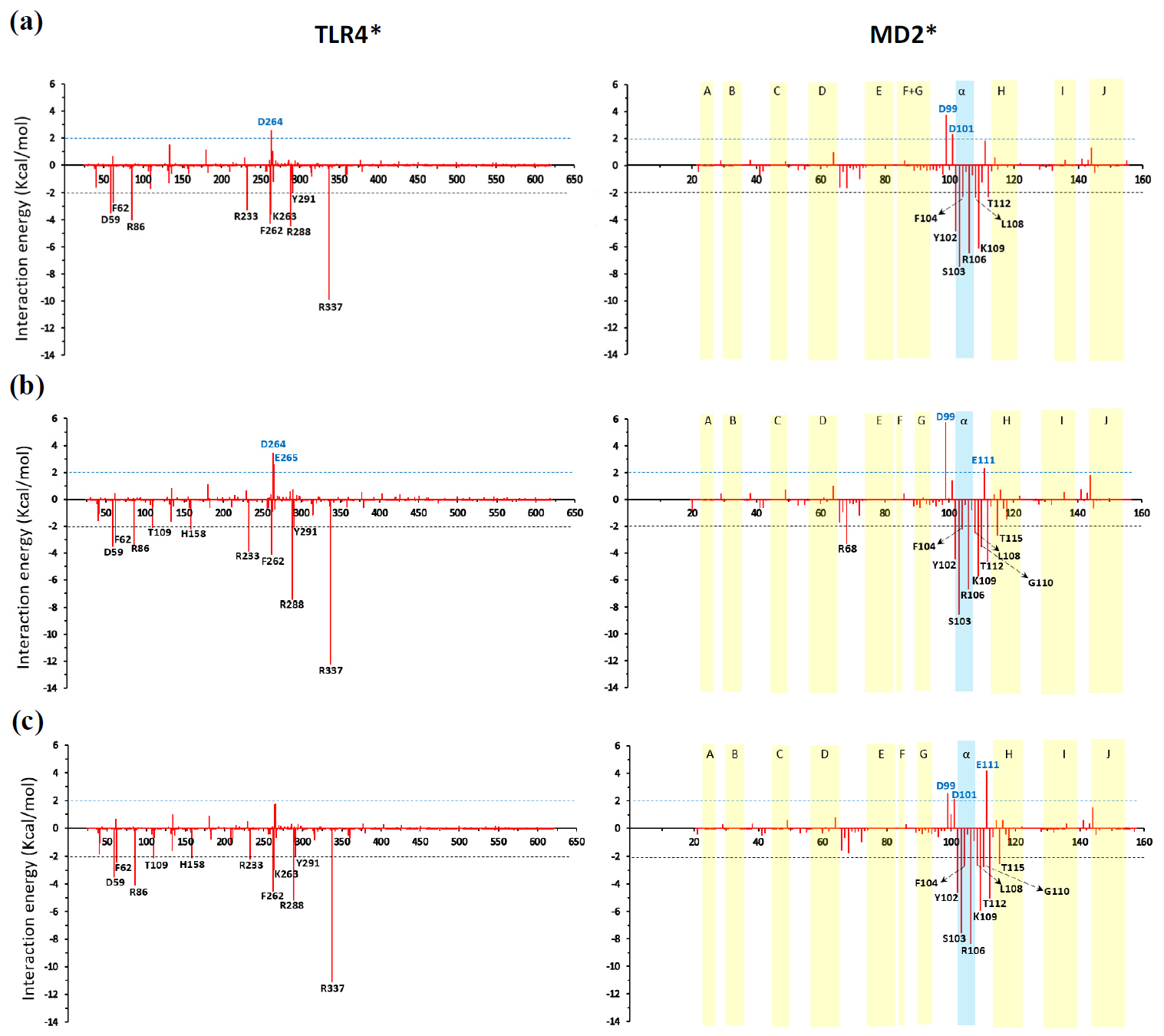

Supplement: S3 Fig — a) the ligand-free (TLR4-MD2)2 tetramer, b) the lipopolysaccharide (LPS)-bound (TLR4-MD2)2 tetramer, and c) the neoseptin3-bound (TLR4-MD2)2 tetramer complex. The favorable key residues (lower than -2 kcal/mol) and unfavorable residues (greater than 2 kcal/mol) are shown in black and blue, respectively. (TIF) [file pcbi.1007228.s003.tif]

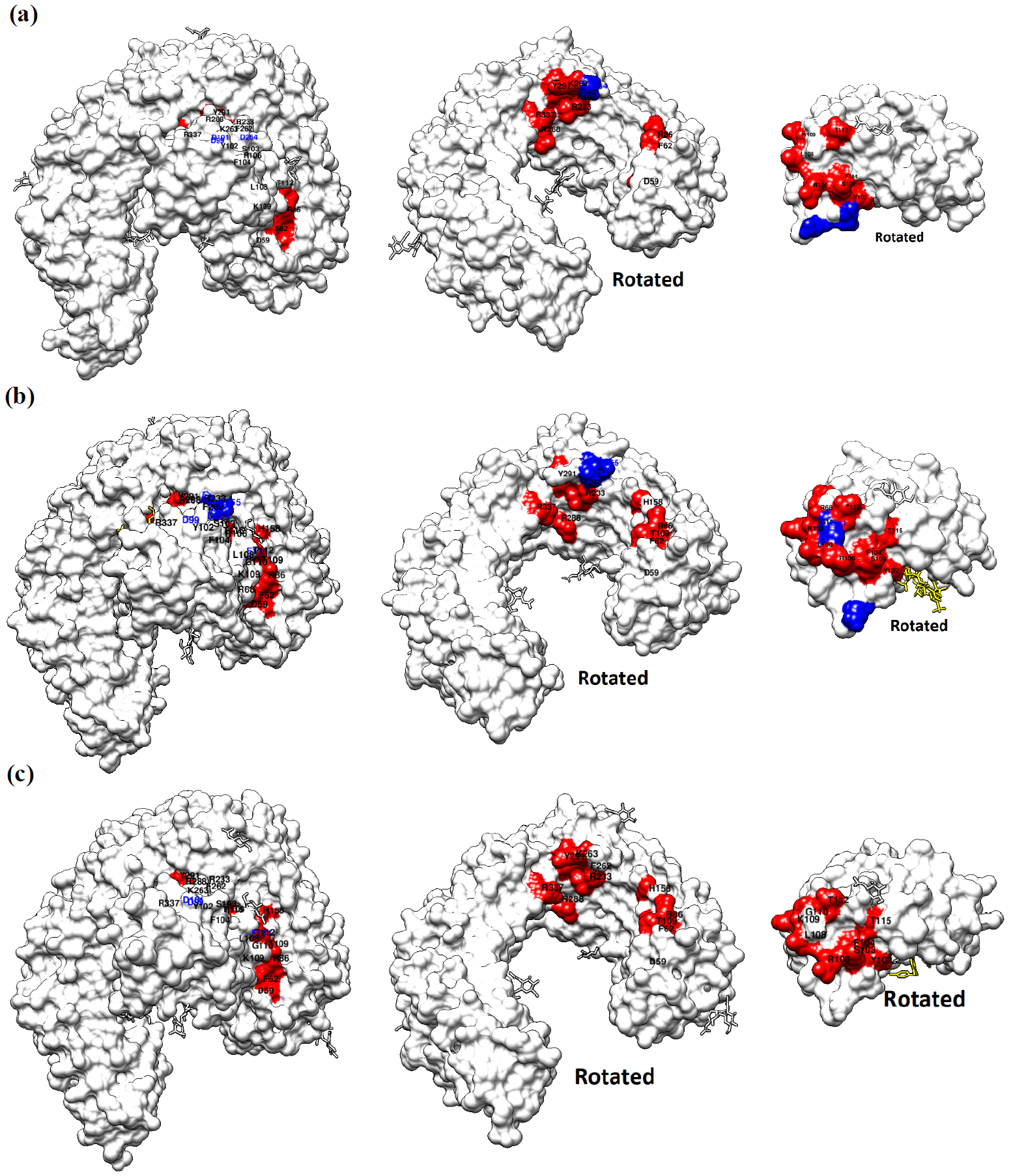

Supplement: S4 Fig — a) the ligand-free (TLR4-MD2)2 tetramer, b) the lipopolysaccharide (LPS)-bound (TLR4-MD2)2 tetramer, and c) the neoseptin3-bound (TLR4-MD2)2 tetramer complex. The favorable and unfavorable residues are colored in red and blue, respectively and the ligands (LPS or neoseptin3) are colored in yellow. The TLR4* and MD*2 monomers are rotated for the best view. (TIF) [file pcbi.1007228.s004.tif]

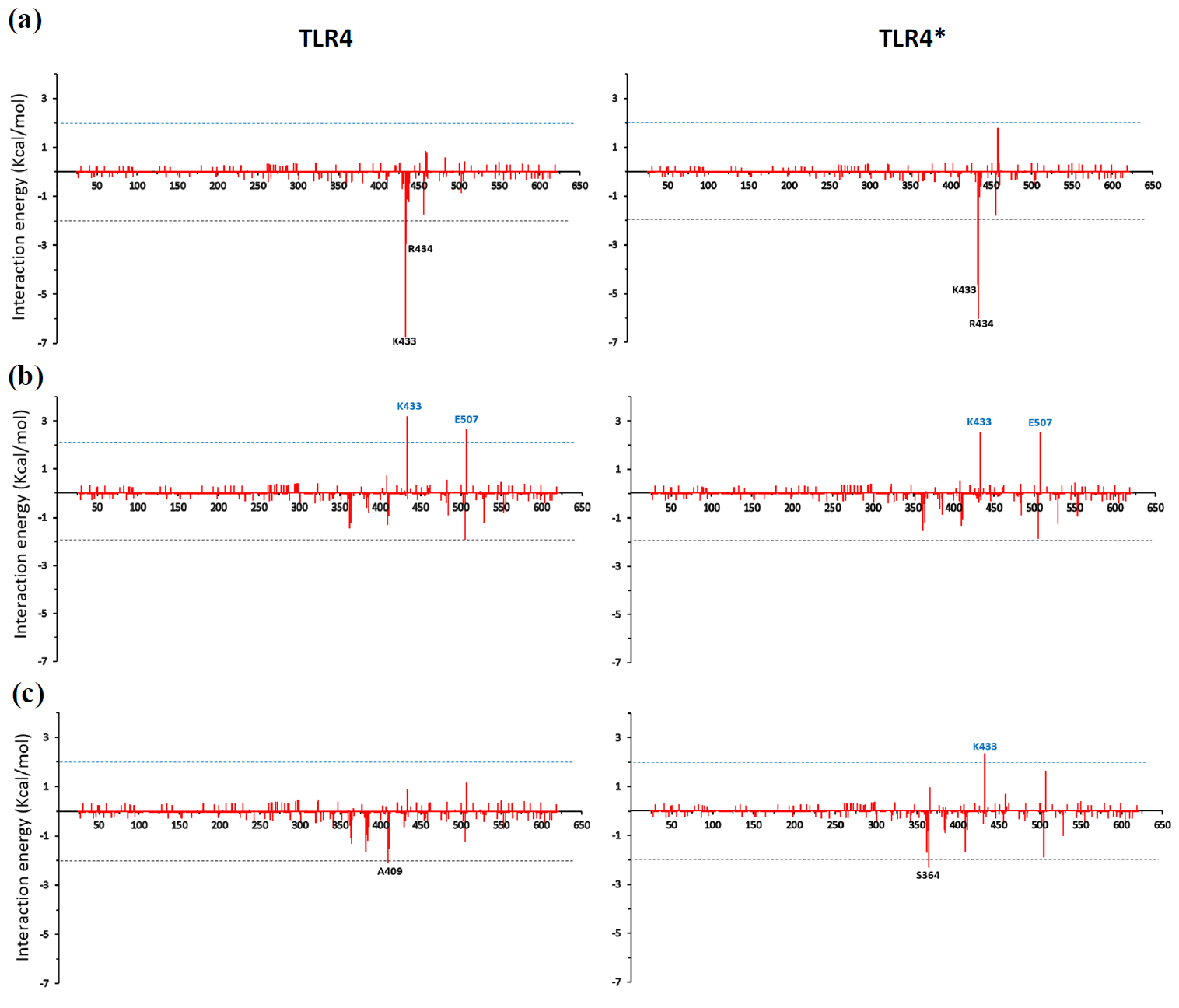

Supplement: S5 Fig — a) the ligand-free (TLR4-MD2)2 tetramer, b) the lipopolysaccharide (LPS)-bound (TLR4-MD2)2 tetramer, and c) the neoseptin3-bound (TLR4-MD2)2 tetramer complex. The favorable key residues (lower than -2 kcal/mol) and unfavorable residues (greater than 2 kcal/mol) are shown in black and blue, respectively. (TIF) [file pcbi.1007228.s005.tif]

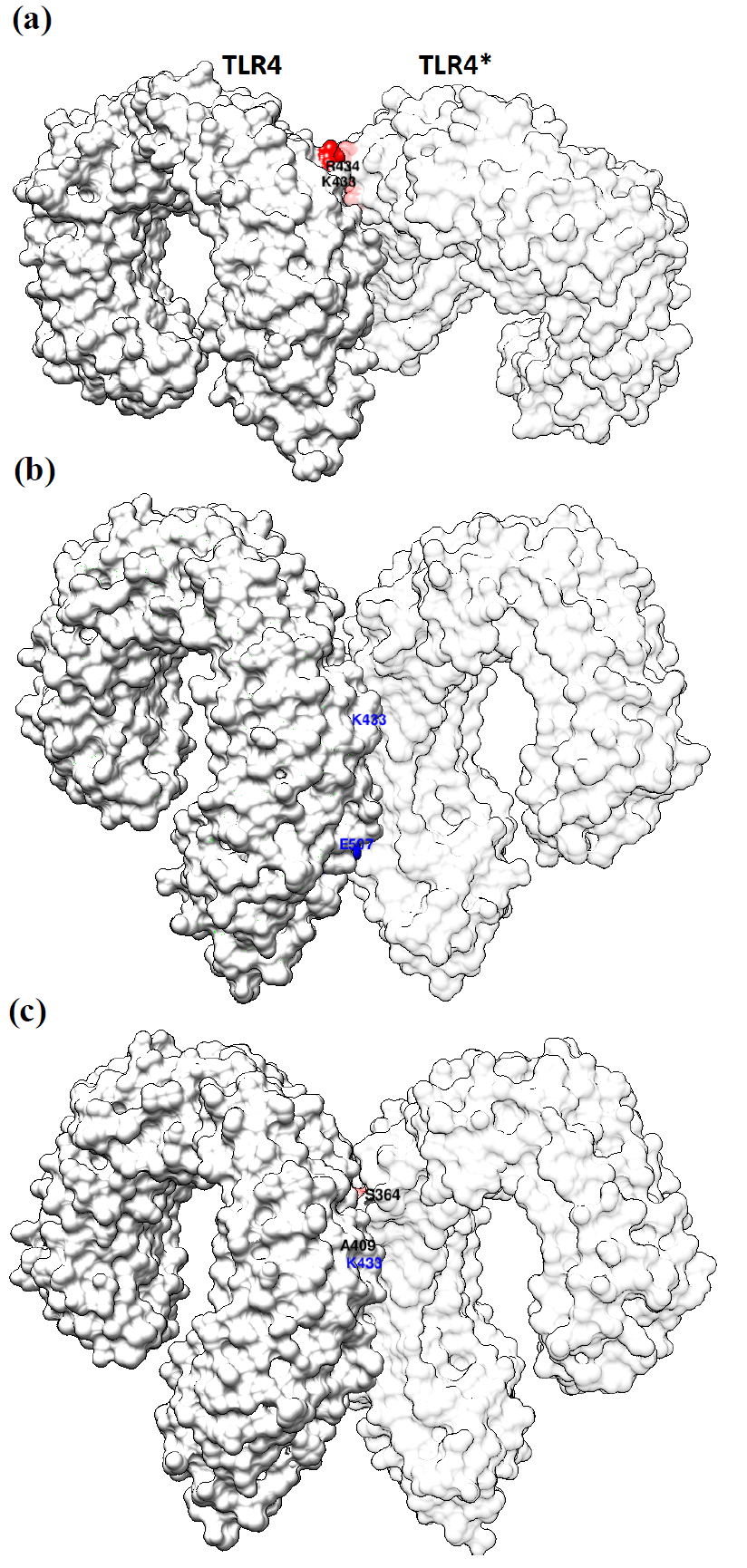

Supplement: S6 Fig — a) the ligand-free (TLR4-MD2)2 tetramer, b) the lipopolysaccharide (LPS)-bound (TLR4-MD2)2 tetramer, and c) the neoseptin3-bound (TLR4-MD2)2 tetramer complex. The favorable and unfavorable residues are colored in red and blue, respectively. The TLR4* monomer has been shown in a more transparent representation. (TIF) [file pcbi.1007228.s006.tif]

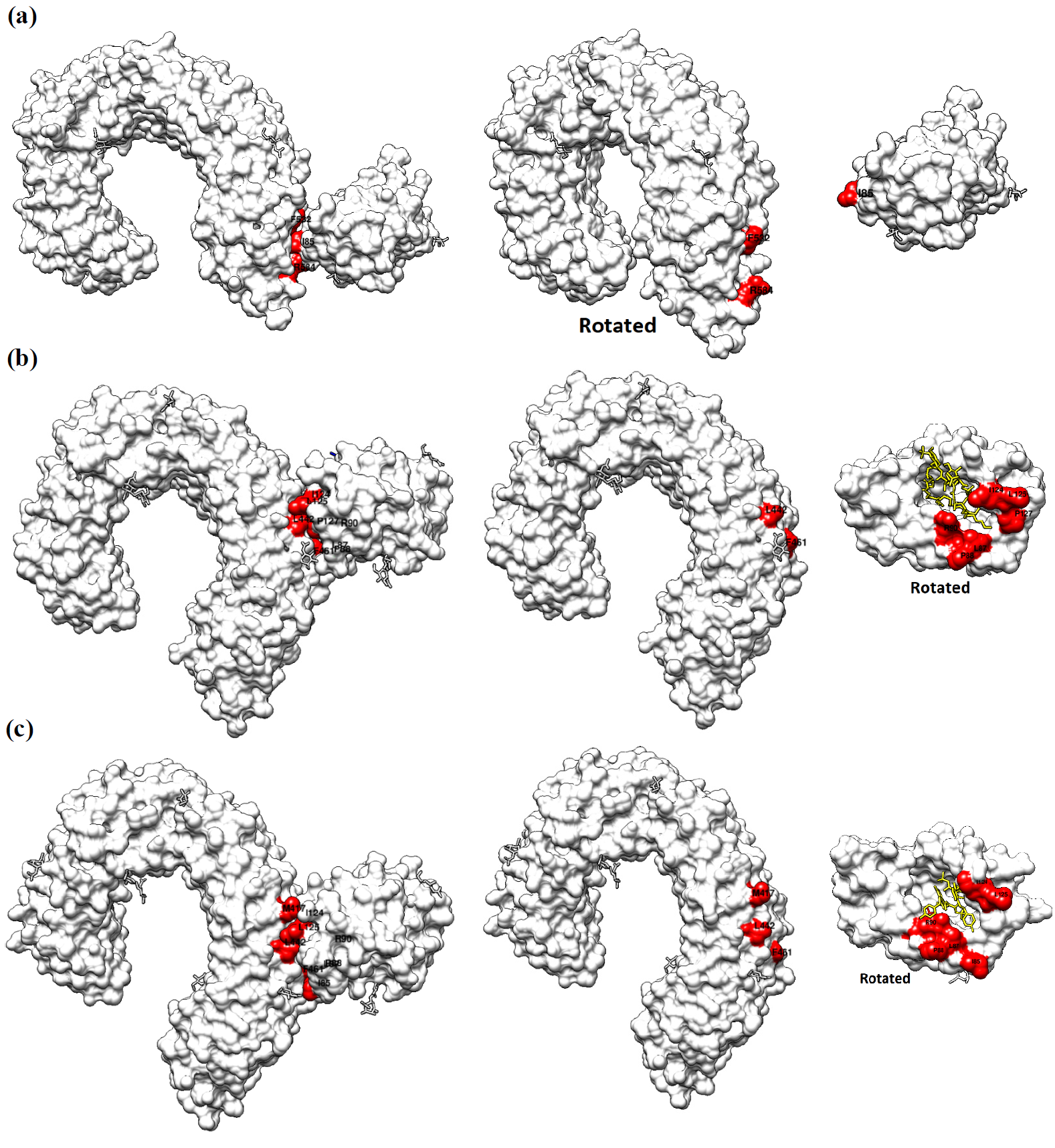

Supplement: S7 Fig — a) the ligand-free (TLR4-MD2)2 tetramer, b) the lipopolysaccharide (LPS)-bound (TLR4-MD2)2 tetramer, and c) the neoseptin3-bound (TLR4-MD2)2 tetramer complex. The favorable and unfavorable residues are colored in red and blue, respectively and the ligands (LPS or neoseptin3) are colored in yellow. The TLR4 and MD*2 monomers are rotated for the best view. (TIF) [file pcbi.1007228.s007.tif]

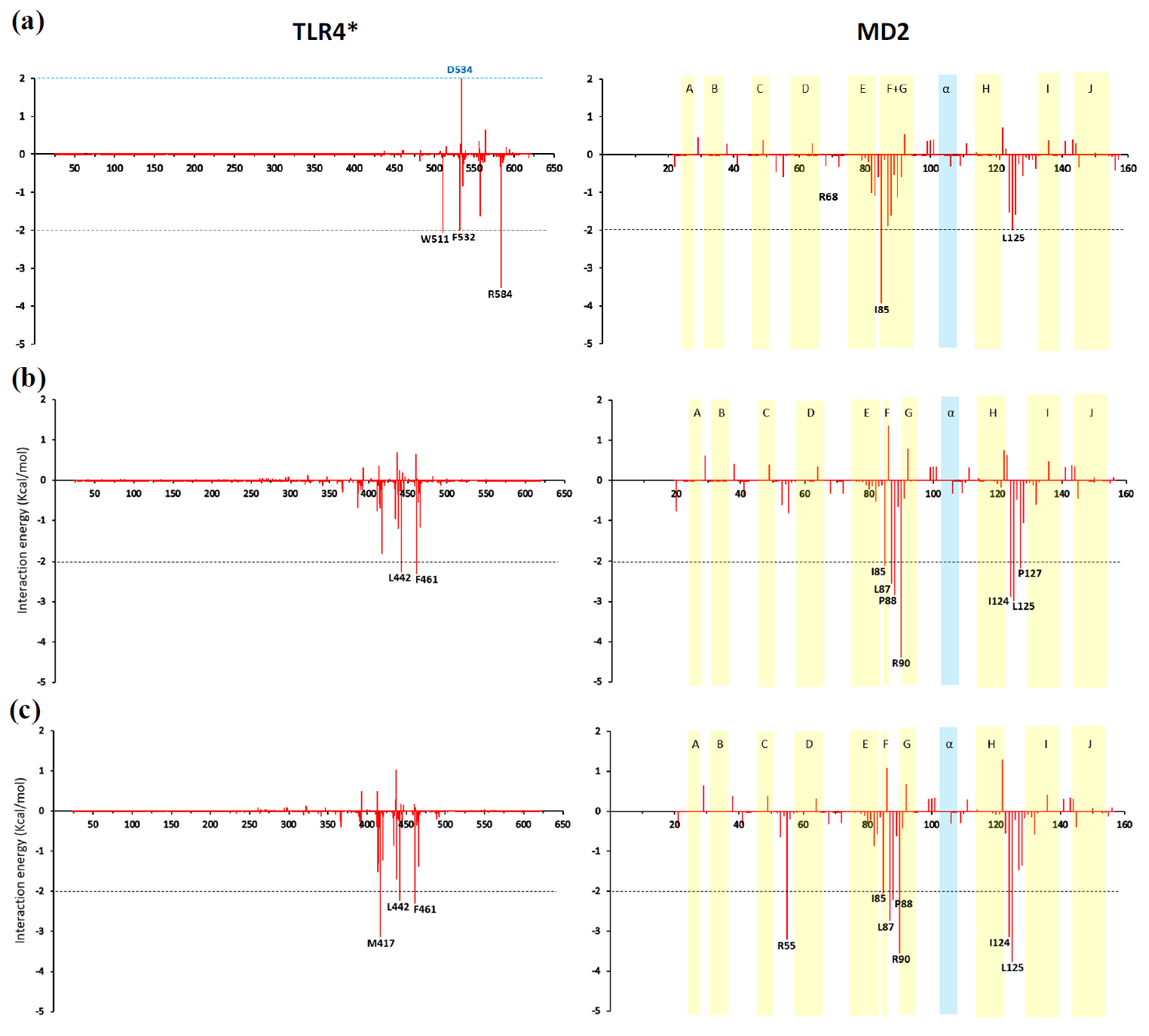

Supplement: S8 Fig — a) the ligand-free (TLR4-MD2)2 tetramer, b) the lipopolysaccharide (LPS)-bound (TLR4-MD2)2 tetramer, and c) the neoseptin3-bound (TLR4-MD2)2 tetramer complex. The favorable key residues (lower than -2 kcal/mol) and unfavorable residues (greater than 2 kcal/mol) are shown in black and blue, respectively. (TIF) [file pcbi.1007228.s008.tif]

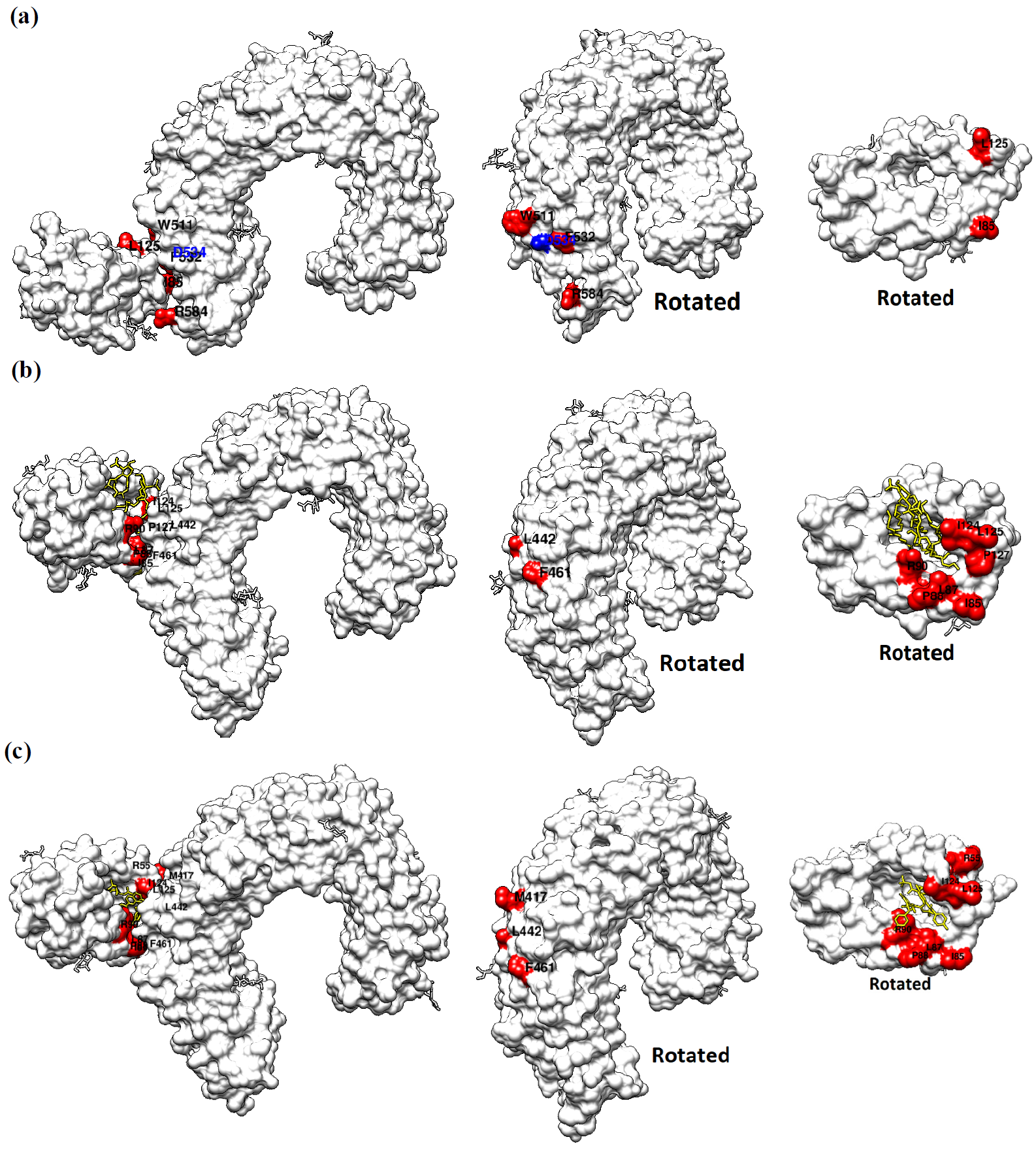

Supplement: S9 Fig — a) the ligand-free (TLR4-MD2)2 tetramer, b) the lipopolysaccharide (LPS)-bound (TLR4-MD2)2 tetramer, and c) the neoseptin3-bound (TLR4-MD2)2 tetramer complex. The favorable and unfavorable residues are colored in red and blue, respectively and the ligands (LPS or neoseptin3) are colored in yellow. The TLR4* and MD2 monomers are rotated for the best view. (TIF) [file pcbi.1007228.s009.tif]

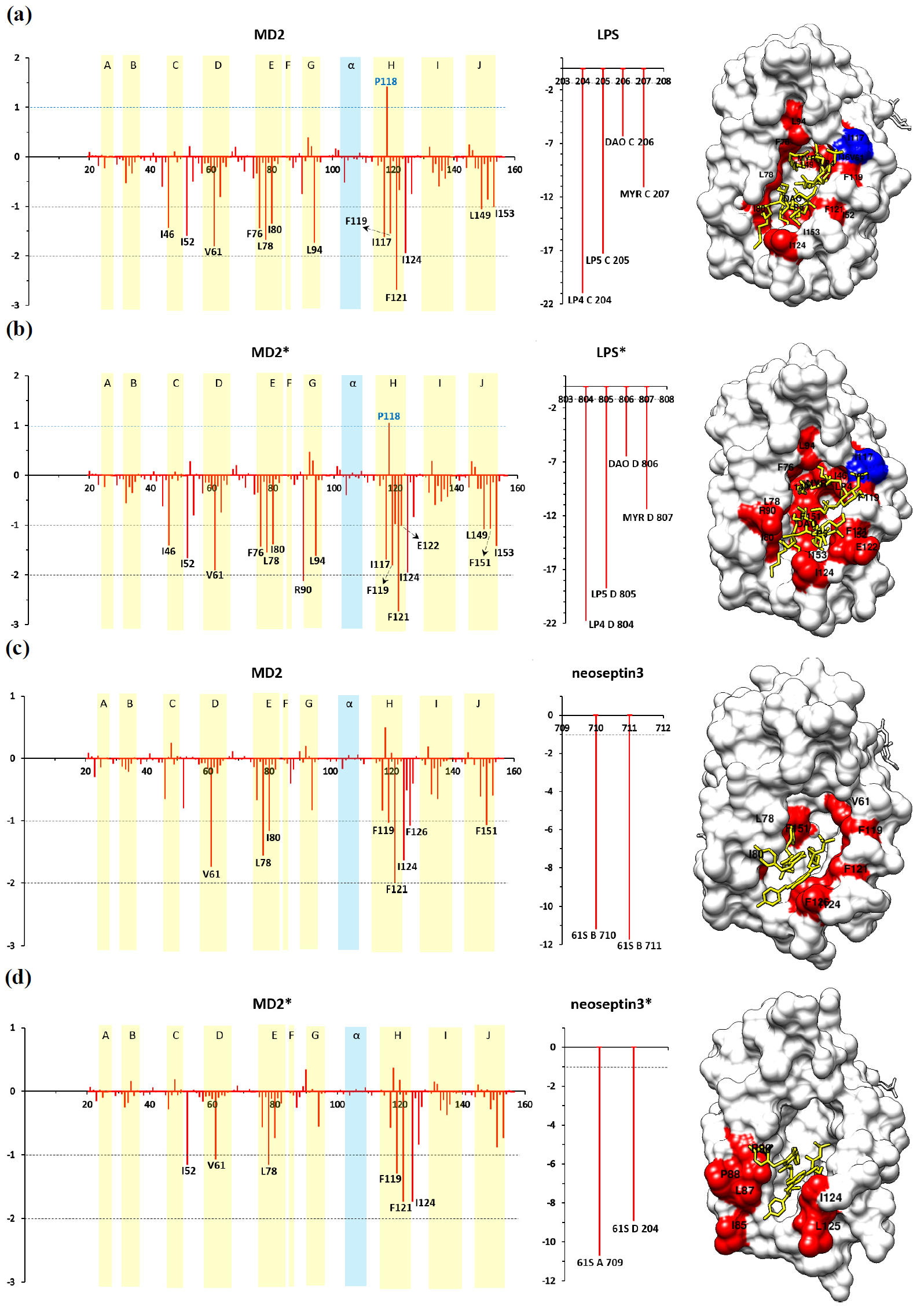

Supplement: S10 Fig — The per residue energy contribution spectrums of MD2, MD2* and ligands (LPS, neoseptin3) in the MD2/ligand or MD2*/ligand interface of a and b) the lipopolysaccharide (LPS)-bound (TLR4-MD2)2 tetramer complex, c and d) the neoseptin3-bound (TLR4-MD2)2 tetramer complex. The favorable key residues (lower than -2 and -1 kcal/mol) and unfavorable residues (greater than 1 kcal/mol) are shown in black and blue, respectively. The Illustration of these residues are shown next to each spectrum. The favorable and unfavorable residues are colored in red and blue, respectively and the ligands (LPS or neoseptin3) are colored in yellow. (TIF) [file pcbi.1007228.s010.tif]
