# Supplementary material for: Key residues in TLR4-MD2 tetramer formation identified by free energy simulations
Source: PLoS Comput Biol. 2019 Oct 14;15(10):e1007228. doi: 10.1371/journal.pcbi.1007228 (PMC6812856; doi:10.1371/journal.pcbi.1007228)
Supplement: S1 Table — Trajectories in each complex are identified with a number from 1 to 4 (#) and their average is denoted by ‘1–4’ as shaded in light grey. The values in parenthesis are standard deviation. (PDF) [file pcbi.1007228.s011.pdf]

Table S1. The C_α root mean-square deviation (RMSD) of ligand-free TLR4-MD2 heterodimer, $(TLR4\text{-}MD2)_2$ tetramer, lipopolysaccharide (LPS)-bound $(TLR4\text{-}MD2)_2$ tetramer, and neoseptin3-bound $(TLR4\text{-}MD2)_2$ tetramer complexes are averaged over the last 1.0 µs of each trajectory. Trajectories in each complex are identified with a number from 1 to 4 (#) and their average is denoted by '1-4' as shaded in light grey. The values in parenthesis are standard deviation.

**Complex**	**#**	***RMSD***
(TLR4-MD2)	1	**3.82** (0.95)
(TLR4-MD2)	2	**2.81** (0.53)
(TLR4-MD2)	3	**3.40** (0.54)
(TLR4-MD2)	4	**4.05** (0.62)
(TLR4-MD2)	1-4	**3.52** (0.55)
$(TLR4\text{-}MD2)_2$	1	**4.33** (0.46)
$(TLR4\text{-}MD2)_2$	2	**3.07** (0.32)
$(TLR4\text{-}MD2)_2$	3	**4.24** (0.83)
$(TLR4\text{-}MD2)_2$	4	**4.10** (0.46)
$(TLR4\text{-}MD2)_2$	1-4	**3.93** (0.58)
$(TLR4\text{-}MD2\text{-}LPS)_2$	1	**2.44** (0.36)
$(TLR4\text{-}MD2\text{-}LPS)_2$	2	**2.69** (0.38)
$(TLR4\text{-}MD2\text{-}LPS)_2$	3	**2.68** (0.49)
$(TLR4\text{-}MD2\text{-}LPS)_2$	4	**2.25** (0.28)
$(TLR4\text{-}MD2\text{-}LPS)_2$	1-4	**2.51** (0.21)
$(TLR4\text{-}MD2\text{-}neoseptin3)_2$	1	**2.86** (0.40)
$(TLR4\text{-}MD2\text{-}neoseptin3)_2$	2	**3.13** (0.28)
$(TLR4\text{-}MD2\text{-}neoseptin3)_2$	3	**2.87** (0.39)
$(TLR4\text{-}MD2\text{-}neoseptin3)_2$	4	**2.88** (0.42)
$(TLR4\text{-}MD2\text{-}neoseptin3)_2$	1-4	**2.93** (0.13)
