# Supplementary material for: Key residues in TLR4-MD2 tetramer formation identified by free energy simulations
Source: PLoS Comput Biol. 2019 Oct 14;15(10):e1007228. doi: 10.1371/journal.pcbi.1007228 (PMC6812856; doi:10.1371/journal.pcbi.1007228)
Supplement: S3 Table — (PDF) [file pcbi.1007228.s013.pdf]

Table S3. The binding free energies (ΔG) in kcal/mol computed by both molecular mechanics generalized Born surface area (MM-GBSA) and molecular mechanics Poisson-Boltzmann surface area (MM-PBSA) methods at TLR4*/MD2* interface.

				Generalized Born (GB)			Poisson-Boltzmann (PB)		
Complex	#	ΔE_{MM}		ΔG_{sol}		ΔG	ΔG_{sol}		ΔG
		ΔE_{ele}	ΔE_{vdw}	ΔG_{pol}	ΔG_{nonpol}		ΔG_{pol}	ΔG_{nonpol}	
$(TLR4\text{-}MD2)_2$	1	**-401.86** (49.13)	**-96.31** (6.34)	**433.13** (45.34)	**-14.79** (0.77)	**-79.82** (9.34)	**415.09** (44.74)	**-11.18** (0.47)	**-94.25** (10.69)
$(TLR4\text{-}MD2)_2$	2	**-415.29** (72.99)	**-105.82** (7.04)	**450.54** (65.60)	**-16.07** (0.82)	**-86.63** (11.26)	**437.15** (67.02)	**-11.75** (0.58)	**-95.69** (12.47)
$(TLR4\text{-}MD2)_2$	3	**-470.44** (52.58)	**-104.50** (6.58)	**492.72** (48.24)	**-16.83** (0.59)	**-99.03** (10.67)	**481.87** (47.50)	**-12.04** (0.40)	**-105.08** (11.19)
$(TLR4\text{-}MD2)_2$	4	**-492.07** (66.78)	**-95.80** (6.99)	**517.40** (62.20)	**-15.27** (0.90)	**-85.73** (10.09)	**500.28** (61.22)	**-11.52** (0.63)	**-99.10** (12.00)
$(TLR4\text{-}MD2)_2$	1-4	**-444.92** (43.23)	**-100.61** (5.29)	**473.45** (38.53)	**-15.74** (0.90)	**-87.80** (8.07)	**458.60** (39.29)	**-11.62** (0.36)	**-98.53** (4.82)
$(TLR4\text{-}MD2\text{-}LPS)_2$	1	**-567.11** (48.40)	**-116.14** (7.22)	**592.14** (43.89)	**-18.54** (0.70)	**-109.63** (10.42)	**583.25** (44.25)	**-12.73** (0.42)	**-112.71** (11.60)
$(TLR4\text{-}MD2\text{-}LPS)_2$	2	**-547.10** (53.04)	**-120.28** (7.77)	**580.17** (47.45)	**-18.72** (0.97)	**-105.92** (12.65)	**567.11** (46.63)	**-13.11** (0.74)	**-113.36** (15.97)
$(TLR4\text{-}MD2\text{-}LPS)_2$	3	**-549.78** (63.52)	**-115.51** (8.24)	**584.28** (60.35)	**-17.82** (1.22)	**-98.81** (12.27)	**566.30** (62.55)	**-12.42** (0.79)	**-111.39** (14.22)
$(TLR4\text{-}MD2\text{-}LPS)_2$	4	**-580.18** (70.14)	**-112.56** (7.65)	**599.07** (61.85)	**-18.14** (0.90)	**-111.80** (13.94)	**586.04** (59.56)	**-12.80** (0.48)	**-119.49** (17.09)
$(TLR4\text{-}MD2\text{-}LPS)_2$	1-4	**-561.04** (15.54)	**-116.12** (3.18)	**588.92** (8.40)	**-18.31** (0.40)	**-106.54** (5.70)	**575.68** (10.43)	**-12.77** (0.28)	**-114.24** (3.60)
$(TLR4\text{-}MD2\text{-}neoseptin3)_2$	1	**-483.32** (43.87)	**-106.69** (7.47)	**510.33** (40.04)	**-16.39** (0.93)	**-96.06** (11.58)	**491.43** (40.10)	**-11.62** (0.45)	**-110.19** (11.64)
$(TLR4\text{-}MD2\text{-}neoseptin3)_2$	2	**-505.96** (55.91)	**-107.56** (7.17)	**530.87** (53.74)	**-16.52** (0.72)	**-99.15** (9.15)	**513.66** (52.82)	**-11.70** (0.51)	**-111.55** (11.48)
$(TLR4\text{-}MD2\text{-}neoseptin3)_2$	3	**-588.88** (62.03)	**-109.72** (7.24)	**605.98** (58.38)	**-17.65** (0.87)	**-110.24** (9.82)	**589.74** (58.38)	**-12.53** (0.60)	**-121.37** (12.54)
$(TLR4\text{-}MD2\text{-}neoseptin3)_2$	4	**-501.90** (50.60)	**-106.88** (7.35)	**524.92** (46.88)	**-16.70** (0.68)	**-100.54** (9.38)	**511.95** (46.12)	**-11.66** (0.36)	**-108.47** (11.21)
$(TLR4\text{-}MD2\text{-}neoseptin3)_2$	1-4	**-520.02** (46.96)	**-107.71** (1.39)	**543.03** (42.85)	**-16.82** (0.57)	**-101.50** (6.12)	**526.70** (43.23)	**-11.88** (0.44)	**-112.90** (5.79)
