# Supplementary material for: Key residues in TLR4-MD2 tetramer formation identified by free energy simulations
Source: PLoS Comput Biol. 2019 Oct 14;15(10):e1007228. doi: 10.1371/journal.pcbi.1007228 (PMC6812856; doi:10.1371/journal.pcbi.1007228)
Supplement: S4 Table — (PDF) [file pcbi.1007228.s014.pdf]

Table S4. The binding free energies (ΔG) in kcal/mol computed by both molecular mechanics generalized Born surface area (MM-GBSA) and molecular mechanics Poisson-Boltzmann surface area (MM-PBSA) methods at TLR4/TLR4* interface.

				Generalized Born (GB)			Poisson-Boltzmann (PB)		
Complex	#	ΔE_{MM}		ΔG_{sol}		ΔG	ΔG_{sol}		ΔG
		ΔE_{ele}	ΔE_{vdw}	ΔG_{pol}	ΔG_{nonpol}		ΔG_{pol}	ΔG_{nonpol}	
$(TLR4\text{-}MD2)_2$	1	**-147.29** (66.89)	**-44.66** (5.57)	**186.86** (63.12)	**-8.38** (0.85)	**-13.44** (8.31)	**161.34** (62.61)	**-7.27** (0.59)	**-37.86** (11.92)
$(TLR4\text{-}MD2)_2$	2	**-78.99** (35.49)	**-42.97** (8.41)	**115.25** (36.00)	**-7.44** (1.27)	**-14.13** (6.74)	**89.17** (36.79)	**-6.25** (0.93)	**-39.01** (9.77)
$(TLR4\text{-}MD2)_2$	3	**-47.99** (29.34)	**-31.50** (5.81)	**70.24** (28.23)	**-5.38** (0.62)	**-14.60** (4.58)	**48.45** (27.68)	**-4.49** (0.48)	**-35.50** (6.21)
$(TLR4\text{-}MD2)_2$	4	**-228.82** (56.02)	**-50.39** (8.15)	**259.70** (52.92)	**-9.03** (1.32)	**-28.51** (10.42)	**239.53** (52.13)	**-7.39** (0.85)	**-47.04** (13.89)
$(TLR4\text{-}MD2)_2$	1-4	**-125.77** (80.25)	**-42.38** (7.92)	**158.01** (83.08)	**-7.56** (1.59)	**-17.67** (7.24)	**134.62** (84.09)	**-6.35** (1.34)	**-39.85** (5.01)
$(TLR4\text{-}MD2\text{-}LPS)_2$	1	**258.48** (40.74)	**-66.02** (12.20)	**-183.62** (45.31)	**-8.44** (1.89)	**0.41** (9.09)	**-210.06** (44.72)	**-7.86** (1.35)	**-25.45** (10.80)
$(TLR4\text{-}MD2\text{-}LPS)_2$	2	**235.55** (51.41)	**-72.36** (10.04)	**-154.92** (52.59)	**-9.98** (1.50)	**-1.69** (9.70)	**-181.06** (51.49)	**-9.04** (0.97)	**-26.89** (12.54)
$(TLR4\text{-}MD2\text{-}LPS)_2$	3	**251.44** (42.44)	**-65.73** (9.10)	**-172.15** (41.01)	**-8.93** (1.49)	**4.64** (9.55)	**-199.63** (41.30)	**-8.41** (0.99)	**-22.31** (11.39)
$(TLR4\text{-}MD2\text{-}LPS)_2$	4	**223.79** (44.69)	**-75.54** (8.99)	**-142.75** (44.48)	**-10.26** (1.26)	**-4.75** (9.57)	**-171.05** (43.93)	**-9.13** (0.84)	**-31.93** (12.35)
$(TLR4\text{-}MD2\text{-}LPS)_2$	1-4	**242.32** (15.64)	**-69.91** (4.84)	**-163.36** (18.11)	**-9.40** (0.86)	**-0.35** (3.94)	**-190.45** (17.64)	**-8.61** (0.59)	**-26.65** (4.01)
$(TLR4\text{-}MD2\text{-}neoseptin3)_2$	1	**276.68** (32.65)	**-71.64** (6.42)	**-201.17** (32.90)	**-8.65** (0.98)	**-4.77** (6.25)	**-231.08** (32.95)	**-7.65** (0.80)	**-33.67** (7.93)
$(TLR4\text{-}MD2\text{-}neoseptin3)_2$	2	**195.57** (51.16)	**-91.74** (8.11)	**-108.87** (50.33)	**-12.63** (1.21)	**-17.65** (9.71)	**-139.57** (50.43)	**-10.57** (0.77)	**-46.29** (12.39)
$(TLR4\text{-}MD2\text{-}neoseptin3)_2$	3	**240.33** (56.26)	**-80.70** (12.34)	**-160.99** (59.32)	**-10.42** (2.15)	**-11.77** (10.53)	**-190.45** (59.26)	**-8.92** (1.53)	**-39.73** (11.59)
$(TLR4\text{-}MD2\text{-}neoseptin3)_2$	4	**255.73** (45.09)	**-80.25** (10.65)	**-173.19** (48.01)	**-10.23** (1.77)	**-7.93** (9.15)	**-197.95** (47.12)	**-8.87** (1.35)	**-31.33** (11.77)
$(TLR4\text{-}MD2\text{-}neoseptin3)_2$	1-4	**242.08** (34.40)	**-81.08** (8.24)	**-161.06** (38.64)	**-10.48** (1.64)	**-10.53** (5.54)	**-189.76** (37.83)	**-9.00** (1.20)	**-37.76** (6.70)
