# Supplementary material for: Key residues in TLR4-MD2 tetramer formation identified by free energy simulations
Source: PLoS Comput Biol. 2019 Oct 14;15(10):e1007228. doi: 10.1371/journal.pcbi.1007228 (PMC6812856; doi:10.1371/journal.pcbi.1007228)
Supplement: S5 Table — (PDF) [file pcbi.1007228.s015.pdf]

Table S5. The binding free energies (ΔG) in kcal/mol computed by both molecular mechanics generalized Born surface area (MM-GBSA) and molecular mechanics Poisson-Boltzmann surface area (MM-PBSA) methods at TLR4/MD2* interface.

				Generalized Born (GB)			Poisson-Boltzmann (PB)		
Complex	#	ΔE_{MM}		ΔG_{sol}		ΔG	ΔG_{sol}		ΔG
		ΔE_{ele}	ΔE_{vdw}	ΔG_{pol}	ΔG_{nonpol}		ΔG_{pol}	ΔG_{nonpol}	
$(TLR4\text{-}MD2)_2$	1	**4.47** (36.90)	**-38.74** (7.28)	**23.55** (33.15)	**-5.00** (0.96)	**-15.71** (6.82)	**12.09** (34.06)	**-4.81** (0.76)	**-26.97** (7.54)
$(TLR4\text{-}MD2)_2$	2	**-60.50** (47.75)	**-55.51** (11.58)	**93.04** (45.30)	**-7.30** (1.68)	**-30.26** (11.76)	**83.81** (46.33)	**-6.79** (1.26)	**-38.97** (11.35)
$(TLR4\text{-}MD2)_2$	3	**-50.35** (39.49)	**-33.67** (5.78)	**70.99** (37.36)	**-4.60** (0.76)	**-17.61** (6.11)	**57.80** (37.36)	**-4.34** (0.66)	**-30.54** (6.78)
$(TLR4\text{-}MD2)_2$	4	**-33.03** (50.61)	**-33.99** (12.09)	**53.77** (51.33)	**-4.83** (1.89)	**-18.07** (9.88)	**46.29** (53.04)	**-4.39** (1.51)	**-25.11** (9.51)
$(TLR4\text{-}MD2)_2$	1-4	**-34.85** (28.56)	**-40.48** (10.29)	**60.34** (29.32)	**-5.43** (1.26)	**-20.41** (6.64)	**50.00** (29.75)	**-5.08** (1.16)	**-30.40** (6.14)
$(TLR4\text{-}MD2\text{-}LPS)_2$	1	**-120.80** (34.84)	**-78.00** (5.40)	**170.82** (33.87)	**-9.97** (0.90)	**-37.93** (6.26)	**167.08** (33.80)	**-8.02** (0.56)	**-39.71** (8.44)
$(TLR4\text{-}MD2\text{-}LPS)_2$	2	**-142.00** (33.33)	**-73.00** (14.89)	**191.09** (35.17)	**-9.55** (2.01)	**-33.45** (9.91)	**190.02** (39.55)	**-7.70** (1.22)	**-32.67** (7.68)
$(TLR4\text{-}MD2\text{-}LPS)_2$	3	**-264.03** (46.23)	**-72.56** (8.88)	**288.19** (44.52)	**-10.42** (1.13)	**-58.79** (9.13)	**278.58** (44.90)	**-8.27** (0.73)	**-66.25** (9.75)
$(TLR4\text{-}MD2\text{-}LPS)_2$	4	**-142.75** (32.63)	**-72.84** (9.64)	**185.42** (28.66)	**-9.85** (1.35)	**-39.99** (11.06)	**176.64** (28.29)	**-7.96** (0.78)	**-46.87** (11.87)
$(TLR4\text{-}MD2\text{-}LPS)_2$	1-4	**-167.40** (65.22)	**-74.10** (2.61)	**208.88** (53.56)	**-9.95** (0.36)	**-42.54** (11.17)	**203.08** (51.21)	**-7.99** (0.23)	**-46.38** (14.46)
$(TLR4\text{-}MD2\text{-}neoseptin3)_2$	1	**-124.29** (48.80)	**-78.65** (10.16)	**171.30** (46.78)	**-9.92** (1.36)	**-41.55** (11.35)	**159.39** (47.20)	**-8.08** (0.79)	**-51.62** (12.99)
$(TLR4\text{-}MD2\text{-}neoseptin3)_2$	2	**-200.25** (37.33)	**-76.45** (10.49)	**226.49** (32.29)	**-10.87** (1.21)	**-61.07** (9.63)	**220.36** (31.74)	**-8.18** (0.63)	**-64.51** (11.49)
$(TLR4\text{-}MD2\text{-}neoseptin3)_2$	3	**-91.52** (31.26)	**-78.55** (9.09)	**136.69** (30.24)	**-9.77** (1.10)	**-43.13** (9.15)	**129.17** (30.78)	**-7.65** (0.64)	**-48.55** (9.88)
$(TLR4\text{-}MD2\text{-}neoseptin3)_2$	4	**-65.26** (24.58)	**-69.29** (6.16)	**107.35** (23.79)	**-8.82** (0.80)	**-36.01** (5.73)	**101.86** (23.62)	**-7.06** (0.44)	**-39.73** (6.59)
$(TLR4\text{-}MD2\text{-}neoseptin3)_2$	1-4	**-120.33** (58.50)	**-75.74** (4.41)	**160.46** (51.20)	**-9.85** (0.84)	**-45.44** (10.86)	**152.70** (50.86)	**-7.74** (0.51)	**-51.10** (10.26)
