# Supplementary material for: Key residues in TLR4-MD2 tetramer formation identified by free energy simulations
Source: PLoS Comput Biol. 2019 Oct 14;15(10):e1007228. doi: 10.1371/journal.pcbi.1007228 (PMC6812856; doi:10.1371/journal.pcbi.1007228)
Supplement: S6 Table — (PDF) [file pcbi.1007228.s016.pdf]

Table S6. The binding free energies (ΔG) in kcal/mol computed by both molecular mechanics generalized Born surface area (MM-GBSA) and molecular mechanics Poisson-Boltzmann surface area (MM-PBSA) methods at TLR4*/MD2 interface.

				Generalized Born (GB)			Poisson-Boltzmann (PB)		
Complex	#	ΔE_{MM}		ΔG_{sol}		ΔG	ΔG_{sol}		ΔG
		ΔE_{ele}	ΔE_{vdw}	ΔG_{pol}	ΔG_{nonpol}		ΔG_{pol}	ΔG_{nonpol}	
$(TLR4\text{-}MD2)_2$	1	**-130.70** (35.33)	**-54.16** (8.46)	**163.46** (33.19)	**-7.49** (1.25)	**-28.87** (7.62)	**153.52** (34.08)	**-6.99** (1.03)	**-38.30** (8.24)
$(TLR4\text{-}MD2)_2$	2	**-127.25** (48.76)	**-62.64** (8.92)	**167.76** (44.97)	**-8.43** (1.19)	**-32.20** (8.54)	**157.69** (45.50)	**-7.54** (0.72)	**-39.72** (8.53)
$(TLR4\text{-}MD2)_2$	3	**-111.24** (45.27)	**-38.96** (8.76)	**139.91** (42.32)	**-5.43** (1.21)	**-17.19** (7.98)	**127.33** (42.98)	**-5.15** (0.94)	**-28.00** (7.96)
$(TLR4\text{-}MD2)_2$	4	**-100.08** (41.04)	**-53.78** (6.19)	**136.76** (39.61)	**-6.84** (0.89)	**-24.99** (7.11)	**124.68** (39.91)	**-6.09** (0.65)	**-35.25** (8.10)
$(TLR4\text{-}MD2)_2$	1-4	**-117.32** (14.28)	**-52.39** (9.84)	**152.39** (16.31)	**-7.05** (1.26)	**-24.35** (6.36)	**140.81** (17.21)	**-6.44** (1.05)	**-35.32** (5.22)
$(TLR4\text{-}MD2\text{-}LPS)_2$	1	**-129.04** (31.58)	**-75.38** (5.50)	**178.29** (28.47)	**-9.63** (0.69)	**-35.75** (7.07)	**176.32** (28.86)	**-7.67** (0.35)	**-35.75** (8.85)
$(TLR4\text{-}MD2\text{-}LPS)_2$	2	**-139.08** (34.71)	**-71.20** (9.83)	**182.33** (33.32)	**-9.38** (1.27)	**-37.31** (9.05)	**175.18** (35.11)	**-7.63** (0.65)	**-42.72** (9.20)
$(TLR4\text{-}MD2\text{-}LPS)_2$	3	**-153.29** (29.62)	**-67.98** (7.26)	**190.72** (27.97)	**-8.68** (0.98)	**-39.21** (7.73)	**180.47** (28.02)	**-7.33** (0.58)	**-48.11** (9.86)
$(TLR4\text{-}MD2\text{-}LPS)_2$	4	**-274.18** (38.48)	**-78.52** (6.15)	**296.96** (36.13)	**-11.37** (0.81)	**-67.10** (8.50)	**285.85** (35.75)	**-8.82** (0.46)	**-75.67** (9.53)
$(TLR4\text{-}MD2\text{-}LPS)_2$	1-4	**-173.90** (67.59)	**-73.27** (4.63)	**212.96** (55.82)	**-9.77** (1.14)	**-43.96** (15.91)	**204.46** (54.31)	**-7.86** (0.66)	**-50.56** (17.49)
$(TLR4\text{-}MD2\text{-}neoseptin3)_2$	1	**-222.52** (39.26)	**-64.87** (6.93)	**247.09** (36.88)	**-8.95** (0.97)	**-49.23** (8.11)	**235.26** (36.46)	**-7.40** (0.71)	**-59.52** (10.39)
$(TLR4\text{-}MD2\text{-}neoseptin3)_2$	2	**-141.81** (53.77)	**-75.63** (11.47)	**184.43** (51.58)	**-9.65** (1.46)	**-42.65** (9.88)	**173.19** (51.80)	**-7.53** (0.85)	**-51.76** (10.03)
$(TLR4\text{-}MD2\text{-}neoseptin3)_2$	3	**-167.54** (40.85)	**-86.27** (6.39)	**211.26** (37.31)	**-11.45** (0.91)	**-53.98** (8.81)	**201.89** (38.00)	**-8.81** (0.50)	**-60.72** (10.40)
$(TLR4\text{-}MD2\text{-}neoseptin3)_2$	4	**-170.39** (55.75)	**-83.52** (12.27)	**214.76** (51.70)	**-11.14** (1.46)	**-50.27** (12.88)	**204.62** (50.67)	**-8.49** (0.71)	**-57.77** (11.57)
$(TLR4\text{-}MD2\text{-}neoseptin3)_2$	1-4	**-175.57** (33.84)	**-77.57** (9.59)	**215.18** (25.08)	**-10.30** (1.19)	**-48.24** (5.13)	**203.74** (25.37)	**-8.06** (0.70)	**-57.44** (3.98)
