# Supplementary material for: Key residues in TLR4-MD2 tetramer formation identified by free energy simulations
Source: PLoS Comput Biol. 2019 Oct 14;15(10):e1007228. doi: 10.1371/journal.pcbi.1007228 (PMC6812856; doi:10.1371/journal.pcbi.1007228)
Supplement: S7 Table — The free energies in each trajectory of the ligand-free (TLR4-MD2)2 tetramer, lipopolysaccharide (LPS)-bound (TLR4-MD2)2 tetramer, and neoseptin3-bound (TLR4-MD2)2 tetramer complexes are averaged over the 1000 frames from the last 1.0 μs of that trajectory with an interval of 1.0 ns. Trajectories in each complex are identified with a number from 1 to 4 (#) and their average is denoted by ‘1–4’ as shaded in light grey. A negative value is a favorable free energy, while a positive value is an unfavorable. The values in parenthesis are standard deviation. ΔEMM is molecular mechanics free energy which is divided into ΔEele and ΔEvdw representing the contributions from the electrostatic and van der Waals interactions, respectively. ΔGsol is solvation free energy expressed by ΔGpol and ΔGnonpol, the polar and non-polar contributions, respectively. (PDF) [file pcbi.1007228.s017.pdf]

Table S7. The binding free energies (ΔG) in kcal/mol at (TLR4-MD2)/(TLR4*-MD2*) interface computed by both molecular mechanics generalized Born surface area (MM-GBSA) and molecular mechanics Poisson-Boltzmann surface area (MM-PBSA) methods. The free energies in each trajectory of the ligand-free $(TLR4\text{-}MD2)_2$ tetramer, lipopolysaccharide (LPS)-bound $(TLR4\text{-}MD2)_2$ tetramer, and neoseptin3-bound $(TLR4\text{-}MD2)_2$ tetramer complexes are averaged over the 1000 frames from the last 1.0 μs of that trajectory with an interval of 1.0 ns. Trajectories in each complex are identified with a number from 1 to 4 (#) and their average is denoted by '1-4' as shaded in light grey. A negative value is a favorable free energy, while a positive value is an unfavorable. The values in parenthesis are standard deviation. ΔEMM is molecular mechanics free energy which is divided into $\Delta Eele$ and $\Delta Evdw$ representing the contributions from the electrostatic and van der Waals interactions, respectively. $\Delta Gsol$ is solvation free energy expressed by $\Delta Gpol$ and $\Delta Gnonpol$, the polar and non-polar contributions, respectively.

				Generalized Born (GB)			Poisson-Boltzmann (PB)		
**Complex**	**#**	ΔE_{MM}		ΔG_{sol}		ΔG	ΔG_{sol}		ΔG
		ΔE_{ele}	ΔE_{vdw}	ΔG_{pol}	ΔG_{nonpol}		ΔG_{pol}	ΔG_{nonpol}	
$(TLR4\text{-}MD2)_2$	1	**-275.67** (87.78)	**-137.58** (13.02)	**375.87** (82.49)	**-20.87** (1.87)	**-58.23** (12.75)	**329.77** (83.66)	**-19.08** (1.46)	**-102.53** (15.47)
$(TLR4\text{-}MD2)_2$	2	**-266.19** (66.36)	**-161.13** (16.63)	**373.78** (63.72)	**-23.18** (2.19)	**-76.69** (16.56)	**331.21** (64.32)	**-20.58** (1.59)	**-116.66** (18.43)
$(TLR4\text{-}MD2)_2$	3	**-210.04** (67.62)	**-104.15** (12.85)	**280.08** (62.66)	**-15.41** (1.53)	**-49.49** (10.95)	**234.22** (62.54)	**-13.98** (1.22)	**-93.93** (12.46)
$(TLR4\text{-}MD2)_2$	4	**-363.99** (80.16)	**-138.18** (16.62)	**451.11** (80.24)	**-20.70** (2.63)	**-71.74** (15.23)	**413.22** (80.41)	**-17.87** (2.02)	**-106.81** (18.80)
$(TLR4\text{-}MD2)_2$	1-4	**-278.97** (63.65)	**-135.26** (23.46)	**370.21** (70.03)	**-20.04** (3.29)	**-64.04** (12.45)	**327.11** (73.18)	**-17.88** (2.82)	**-104.98** (9.45)
$(TLR4\text{-}MD2\text{-}LPS)_2$	1	**10.39** (65.89)	**-219.44** (13.95)	**161.32** (65.97)	**-28.00** (2.15)	**-75.70** (13.19)	**132.10** (64.93)	**-23.33** (1.44)	**-100.26** (17.71)
$(TLR4\text{-}MD2\text{-}LPS)_2$	2	**-42.30** (66.60)	**-216.60** (15.04)	**213.07** (65.54)	**-28.87** (2.24)	**-74.69** (14.94)	**180.66** (66.48)	**-24.18** (1.35)	**-102.41** (17.18)
$(TLR4\text{-}MD2\text{-}LPS)_2$	3	**-162.23** (76.06)	**-206.31** (13.59)	**300.12** (72.08)	**-27.99** (1.86)	**-96.38** (15.33)	**256.23** (72.21)	**-23.78** (1.14)	**-136.06** (18.09)
$(TLR4\text{-}MD2\text{-}LPS)_2$	4	**-191.27** (61.00)	**-226.94** (15.91)	**333.83** (58.23)	**-31.37** (2.25)	**-115.73** (17.74)	**289.68** (57.84)	**-25.63** (1.43)	**-154.13** (19.93)
$(TLR4\text{-}MD2\text{-}LPS)_2$	1-4	**-96.35** (96.03)	**-217.32** (8.54)	**252.09** (79.06)	**-29.06** (1.60)	**-90.63** (19.49)	**214.67** (71.48)	**-24.23** (1.00)	**-123.22** (26.33)
$(TLR4\text{-}MD2\text{-}neoseptin3)_2$	1	**-62.70** (68.23)	**-215.18** (14.21)	**208.91** (65.78)	**-27.52** (2.03)	**-96.47** (16.15)	**156.79** (65.90)	**-23.13** (1.40)	**-144.20** (18.90)
$(TLR4\text{-}MD2\text{-}neoseptin3)_2$	2	**-132.96** (87.76)	**-243.85** (17.00)	**287.41** (84.77)	**-33.16** (2.35)	**-122.54** (18.54)	**241.70** (86.50)	**-26.28** (1.24)	**-161.37** (20.97)
$(TLR4\text{-}MD2\text{-}neoseptin3)_2$	3	**-9.33** (51.50)	**-245.55** (12.83)	**176.50** (75.18)	**-31.62** (2.05)	**-109.99** (14.73)	**131.95** (75.23)	**-25.30** (1.37)	**-148.21** (17.26)
$(TLR4\text{-}MD2\text{-}neoseptin3)_2$	4	**31.28** (84.48)	**-233.09** (14.66)	**136.04** (83.64)	**-30.19** (2.24)	**-95.94** (16.85)	**98.25** (81.98)	**-24.42** (1.51)	**-127.97** (17.76)
$(TLR4\text{-}MD2\text{-}neoseptin3)_2$	1-4	**-43.43** (71.02)	**-234.42** (13.96)	**202.22** (64.14)	**-30.62** (2.40)	**-106.24** (12.67)	**157.17** (61.25)	**-24.78** (1.34)	**-145.44** (13.76)
