# Supplementary material for: Key residues in TLR4-MD2 tetramer formation identified by free energy simulations
Source: PLoS Comput Biol. 2019 Oct 14;15(10):e1007228. doi: 10.1371/journal.pcbi.1007228 (PMC6812856; doi:10.1371/journal.pcbi.1007228)
Supplement: S8 Table — The values are averaged over the 1000 frames of the combined 4 trajectories of each system. A negative value is a favorable free energy, while a positive value is an unfavorable. The shaded entries are those that have unfavorable free energy contribution of 2.0 kcal/mol or greater. (PDF) [file pcbi.1007228.s018.pdf]

Table S8. The key residues of TLR4 and MD2 identified by per-residue free energy decomposition (kcal/mol) in the TLR4/MD2 and TLR4*/MD2* interfaces of ligand-free TLR4-MD2 heterodimer, (TLR4-MD2)2 tetramer, lipopolysaccharide (LPS)-bound (TLR4-MD2)2 tetramer, and neoseptin3-bound (TLR4-MD2)2 tetramer complexes. The values are averaged over the 1000 frames of the combined 4 trajectories of each system. A negative value is a favorable free energy, while a positive value is an unfavorable. The shaded entries are those that have unfavorable free energy contribution of 2.0 kcal/mol or greater.

		Complex						
		TLR4-MD2	$(TLR4\text{-}MD2)_2$		$(TLR4\text{-}MD2\text{-}LPS)_2$		$(TLR4\text{-}MD2\text{-}neoseptin3)_2$	
		Interface						
Monomer	Residue	TLR4/MD2	TLR4/MD2	TLR4*/MD2*	TLR4/MD2	TLR4*/MD2*	TLR4/MD2	TLR4*/MD2*
TLR4	D59	-3.924	-3.895	-3.536	-3.458	-3.563	-3.765	-3.525
	F62	-2.523	-3.125	-2.758	-2.530	-2.729	-2.603	-2.482
	R86	-3.067	-3.335	-4.060	-3.726	-3.388	-4.115	-4.154
	T109	-1.525	-1.837	-1.687	-2.152	-2.157	-2.272	-2.151
	H158	-0.802	-0.768	-0.640	-1.522	-2.256	-1.887	-2.151
	R233	-3.742	-4.247	-3.283	-3.211	-3.923	-3.424	-2.239
	F262	-4.369	-4.319	-4.272	-4.418	-4.149	-4.099	-4.566
	K263	-3.325	-3.277	-3.629	-1.428	-0.936	-2.875	-3.006
	R288	-5.006	-6.375	-4.468	-7.146	-7.472	-5.757	-5.195
	Y291	-2.020	-2.039	-2.050	-2.156	-2.117	-2.270	-2.045
	R337	-12.805	-12.111	-9.887	-12.621	-12.242	-11.137	-11.109
	E134	2.291	2.241	1.525	1.593	0.815	1.509	0.983
	D264	2.686	3.226	2.556	5.978	3.451	3.598	1.764
	E265	2.508	1.370	1.048	2.440	2.582	1.968	1.810
MD2	R68	-2.493	-4.013	-1.656	-2.151	-3.334	-2.269	-1.773
	Y102	-4.731	-5.158	-4.852	-4.810	-4.471	-5.089	-4.661
	S103	-7.998	-7.953	-7.472	-8.215	-8.549	-7.944	-7.591
	F104	-2.610	-2.603	-2.301	-2.897	-2.235	-2.814	-2.704
	R106	-7.266	-7.521	-6.494	-7.638	-6.759	-6.926	-8.389
	L108	-2.065	-2.166	-2.371	-2.526	-2.536	-2.420	-2.629
	K109	-5.667	-5.564	-6.137	-5.775	-5.724	-6.053	-5.939
	G110	-2.583	-2.663	-1.214	-3.580	-3.587	-2.364	-2.790
	T112	-2.602	-3.099	-2.314	-4.966	-4.667	-4.918	-5.084
	T115	-3.435	-1.679	-0.300	-0.998	-2.708	-1.463	-2.578
	D99	4.151	4.323	3.761	4.997	5.772	4.018	2.554
	D101	2.336	2.006	2.299	2.077	1.413	1.700	2.142
	E111	3.888	4.489	1.836	5.074	2.325	4.511	4.183
Ligand	LPS	–	–	–	-4.75	-4.90	–	–
	neoseptin3	–	–	–	–	–	-4.86	-4.47
