# Supplementary material for: Key residues in TLR4-MD2 tetramer formation identified by free energy simulations
Source: PLoS Comput Biol. 2019 Oct 14;15(10):e1007228. doi: 10.1371/journal.pcbi.1007228 (PMC6812856; doi:10.1371/journal.pcbi.1007228)
Supplement: S9 Table — The values are averaged over the 1000 frames of the combined 4 trajectories of each system. A negative value is a favorable free energy, while a positive value is an unfavorable. The shaded entries are those that have unfavorable free energy contribution of 2.0 kcal/mol or greater. (PDF) [file pcbi.1007228.s019.pdf]

Table S9. The key residues of TLR4 and TLR4* identified by per-residue free energy decomposition (kcal/mol) in the TLR4/TLR4* interface of ligand-free (TLR4-MD2)2 tetramer, lipopolysaccharide (LPS)-bound (TLR4-MD2)2 tetramer, and neoseptin3-bound (TLR4-MD2)2 tetramer complexes. The values are averaged over the 1000 frames of the combined 4 trajectories of each system. A negative value is a favorable free energy, while a positive value is an unfavorable. The shaded entries are those that have unfavorable free energy contribution of 2.0 kcal/mol or greater.

		**Complex**		
		$(TLR4\text{-}MD2)_2$	$(TLR4\text{-}MD2\text{-}LPS)_2$	$(TLR4\text{-}MD2\text{-}neoseptin3)_2$
Monomer	Residue	**TLR4/TLR4* Interface**		
TLR4	S364	-0.034	-1.206	-1.322
	A409	-0.324	-1.309	-2.113
	K433	-6.737	3.183	0.885
	R434	-2.979	-0.282	-0.244
	E507	0.444	2.684	1.154
TLR4*	S364	-0.026	-1.221	-2.314
	A409	-0.247	-1.324	-1.673
	K433	-4.672	2.538	2.376
	R434	-6.015	-0.293	-0.242
	E507	0.386	2.548	1.647
