# Supplementary material for: Key residues in TLR4-MD2 tetramer formation identified by free energy simulations
Source: PLoS Comput Biol. 2019 Oct 14;15(10):e1007228. doi: 10.1371/journal.pcbi.1007228 (PMC6812856; doi:10.1371/journal.pcbi.1007228)
Supplement: S10 Table — The free energies in each of the lipopolysaccharide (LPS)-bound (TLR4-MD2)2 tetramer and neoseptin3-bound (TLR4-MD2)2 tetramer complexes are averaged over the 1000 frames of the combined 4 trajectories. A negative value is a favorable free energy, while a positive value is an unfavorable. The values in parenthesis are standard deviation. ΔEMM is molecular mechanics free energy which is divided into ΔEele and ΔEvdw representing the contributions from the electrostatic and van der Waals interactions, respectively. ΔGsol is solvation free energy expressed by ΔGpol and ΔGnonpol, the polar and non-polar contributions, respectively. (PDF) [file pcbi.1007228.s020.pdf]

Table S10. The binding free energies (ΔG) in kcal/mol between either MD2 or MD2* monomer and the ligands (either LPS or neoseptin3) at either the MD2/ligand or MD2*/ligand interface computed by both molecular mechanics generalized Born surface area (MM-GBSA) and molecular mechanics Poisson-Boltzmann surface area (MM-PBSA) methods. The free energies in each of the lipopolysaccharide (LPS)-bound (TLR4-MD2)$_2$ tetramer and neoseptin3-bound (TLR4-MD2)$_2$ tetramer complexes are averaged over the 1000 frames of the combined 4 trajectories. A negative value is a favorable free energy, while a positive value is an unfavorable. The values in parenthesis are standard deviation. ΔE_{MM} is molecular mechanics free energy which is divided into ΔE_{ele} and ΔE_{vdw} representing the contributions from the electrostatic and van der Waals interactions, respectively. ΔG_{sol} is solvation free energy expressed by ΔG_{pol} and ΔG_{nonpol}, the polar and non-polar contributions, respectively.

					Generalized Born (GB)			Poisson-Boltzmann (PB)		
**Monomer**	**Complex**	**#**	ΔE_{MM}		ΔG_{sol}		ΔG	ΔG_{sol}		ΔG
			ΔE_{ele}	ΔE_{vdw}	ΔG_{pol}	ΔG_{nonpol}		ΔG_{pol}	ΔG_{nonpol}	
**MD2**	(TLR4-MD2-LPS)$_2$	1-4	**-14.50** (17.25)	**-124.26** (8.52)	**47.96** (13.53)	**-17.55** (1.16)	**-108.35** (9.58)	**50.27** (14.60)	**-11.88** (0.58)	**-100.36** (9.40)
**MD2**	(TLR4-MD2-nst3)$_2$	1-4	**-18.81** (12.94)	**-76.25** (7.61)	**47.81** (11.63)	**-10.48** (1.16)	**-57.72** (8.24)	**50.05** (12.60)	**-8.00** (0.55)	**-53.01** (8.10)
**MD2***	(TLR4-MD2-LPS)$_2$	1-4	**-28.93** (22.84)	**-128.43** (10.96)	**59.27** (15.47)	**-18.10** (1.32)	**-116.18** (9.54)	**61.43** (15.78)	**-12.10** (0.54)	**-108.02** (8.76)
**MD2***	(TLR4-MD2-nst3)$_2$	1-4	**-11.27** (13.06)	**-65.14** (12.60)	**38.06** (12.75)	**-8.86** (1.63)	**-47.22** (10.72)	**39.89** (14.21)	**-7.07** (1.02)	**-43.59** (9.92)
